# Determination of thiol metabolites in human urine by stable isotope labeling in combination with pseudo-targeted mass spectrometry analysis

**DOI:** 10.1038/srep21433

**Published:** 2016-02-18

**Authors:** Ping Liu, Chu-Bo Qi, Quan-Fei Zhu, Bi-Feng Yuan, Yu-Qi Feng

**Affiliations:** 1 Key Laboratory of Analytical Chemistry for Biology and Medicine (Ministry of Education), Department of Chemistry, Wuhan University, Wuhan 430072, P.R. China; 2 Department of Pathology, Hubei Cancer Hospital, Wuhan, Hubei 430079, P.R. China

## Abstract

Precursor ion scan and multiple reaction monitoring scan (MRM) are two typical scan modes in mass spectrometry analysis. Here, we developed a strategy by combining stable isotope labeling (IL) with liquid chromatography-mass spectrometry (LC-MS) under double precursor ion scan (DPI) and MRM for analysis of thiols in 5 types of human cancer urine. Firstly, the IL-LC-DPI-MS method was applied for non-targeted profiling of thiols from cancer samples. Compared to traditional full scan mode, the DPI method significantly improved identification selectivity and accuracy. 103 thiol candidates were discovered in all cancers and 6 thiols were identified by their standards. It is worth noting that pantetheine, for the first time, was identified in human urine. Secondly, the IL-LC-MRM-MS method was developed for relative quantification of thiols in cancers compared to healthy controls. All the MRM transitions of light and heavy labeled thiols were acquired from urines by using DPI method. Compared to DPI method, the sensitivity of MRM improved by 2.1–11.3 folds. In addition, the concentration of homocysteine, γ-glutamylcysteine and pantetheine enhanced more than two folds in cancer patients compared to healthy controls. Taken together, the method demonstrated to be a promising strategy for identification and comprehensive quantification of thiols in human urines.

Biological thiols such as cysteine (Cys), homocysteine (HCy), N-acetylcysteine (Nac), glutathione (GSH), cysteinylglycine (CysGly), γ-glutamylcysteine (γ-GluCys), cysteamine (CA), and coenzyme A (CoA) play essential roles in living systems and are involved in a number of biological processes including antioxidant defense network, methionine cycle and protein synthesis^1^. Varying concentrations of some thiols in human plasma and blood are also related to certain cancers, such as breast cancer^2^,^3^, colorectal cancer^4^, and cervical cancer^5^,^6^. In this respect, determination of thiols in biological samples have attracted considerable interest owing to their usefulness as non-invasive diseases diagnostic^7^,^8^ or guidance for follow-up treatment methods^3^,^5^. Many analytical methods have been developed for the analysis of thiols from biological fluids, such as high-performance liquid chromatography (HPLC) and capillary electrophoresis (CE) with ultraviolet (UV)^9^,^10^, fluorescence (FLD)^11^,^12^, electrochemical detection (ED)^13^,^14^ and mass spectrometry (MS) detection^15^,^16^,^17^,^18^. However, only targeted analysis of several thiols was generally performed in these methods.

Recently, stable isotope labeling (IL) strategy has been reported for non-targeted profiling of metabolites with the MS-based platform^19^. The typical IL method introduced a light and heavy isotope tags to the samples, respectively, then mixed the two labeled samples in equal volumes, followed by LC-MS analysis. The MS was normally operated in full scan mode. Extracted peak pairs with characteristic mass difference from the full scan spectra, and the peak pairs with the same retention times and intensities were assigned as candidates^19^. The IL method greatly facilitates the spectral interpretation and metabolite identification because the light/heavy labeled metabolites are always detected as pairs in the mass spectra with characteristic mass difference^20^. MS-based quantification of a large number of metabolites is still challenging due to the fluctuation in MS response and unavailability of isotope internal standards (ISs)^21^,^22^. The IL strategy has also been applied in metabolomics for the relative quantification and/or absolute quantification by calculating the peak intensity ratios of the isotope labeled peak pairs in two comparative samples (or in one sample and standards)^23^. Li *et al.* used ^13^C- and ^12^C-dansyl chloride for the quantification of amine and phenolic hydroxyl metabolites in human urine^20^. Xu *et al.* described a method for steroid hormones quantification in human urine by chemical labeling with 4-(dimethylamino)-benzoic acid (DMBA) and *d*_*4*_-DMBA^24^. However, the full scan mode applied in the aforementioned IL-LC-MS method was not sensitive and accurate enough for quantitative analysis.

For non-targeted profiling of thiols, we recently developed a method based on isotope labeling-high performance liquid chromatography-double precursor ion scan-mass spectrometry (IL-LC-DPI-MS) analysis^25^. We synthesized a pair of isotope labeling reagents (*ω*-bromoacetonylquinolinium bromide, BQB and BQB-*d*_*7*_) for selective thiols labeling. The BQB and BQB-*d*_*7*_ labeled thiols can generate two characteristic product ions at *m/z* 218 and 225, respectively, which therefore can be used for double precursor ion scan (DPI)- in MS analysis. The major advantage of this strategy is that two individual ion chromatograms - are generated; thus, the BQB and BQB-*d*_*7*_ labeled thiols can be clearly distinguished. Extracted characteristic peak pairs from the two precursor ion scan (PI) spectra and assigned them as potential thiol candidates. Compared to the full scan mode, the DPI method can significantly improve the identification sensitivity, selectivity and accuracy. Although, the PI mode in our previous research was highly selective but the quantification sensitivity in MS analysis needs to be improved further.

The MRM mode in LC-MS is widely used in targeted metabolomics^26^,^27^,^28^. It is usually performed on triple quadrupole (QQQ) mass spectrometers, where the first quadrupole isolates the precursor ions, the second quadrupole acts as a collision cell, and the third quadrupole selects the characteristic product ion. A precursor/product pair is referred as a transition. The MRM analysis has good repeatability, sensitivity and broad dynamic range, which therefore can significantly improve the quantification accuracy. However, the limitation of this method is that all the MRM transitions of precursor ions and corresponding product ions should be predefined before sample analysis. The ion pairs are typically acquired from known standards, which restricted the metabolite coverage because it is impossible to obtain a standard for each metabolite. Recently, Xu *et al.* developed a new “pseudo-targeted method” for the serum analysis, where the MRM ion pairs of metabolites were acquired from the real samples through non-targeted analysis using UHPLC/Q-TOF MS^29^. They named the method “pseudo-targeted method” because the metabolites in the MRM were not previously identified. Based on these ion pairs, the MRM mode can be used to detect as many metabolites as possible that are similar to those in the non-targeted metabolic profiling method.

In this work, we developed a novel strategy by combing IL with LC-MS in the DPI and MRM mode for non-targeted profiling and targeted quantitation of thiols from the urine sample of cancer patients. Non-targeted profiling of thiols from the cancer urine by the IL-LC-DPI-MS method provided qualitative information of *m/z* of all the thiol candidates. Then, the IL-LC-MRM-MS method was used for targeted relative quantitation of thiols from cancers and healthy controls. In MRM mode, all the precursor ions of [M]^+^ and [M + 7]^+^ for the MRM transitions of [M]^+^ → 218.1 and [M + 7]^+^ → 225.1 were acquired from the BQB and BQB-*d*_*7*_ labeled samples by using aforementioned DPI method. Our aim was to establish a novel method for non-targeted identification and targeted quantification of thiols in human urine and to investigate the thiol changes in cancers compared to healthy controls and to find thiols as potential cancer biomarkers.

## Results

**Figure 1:**
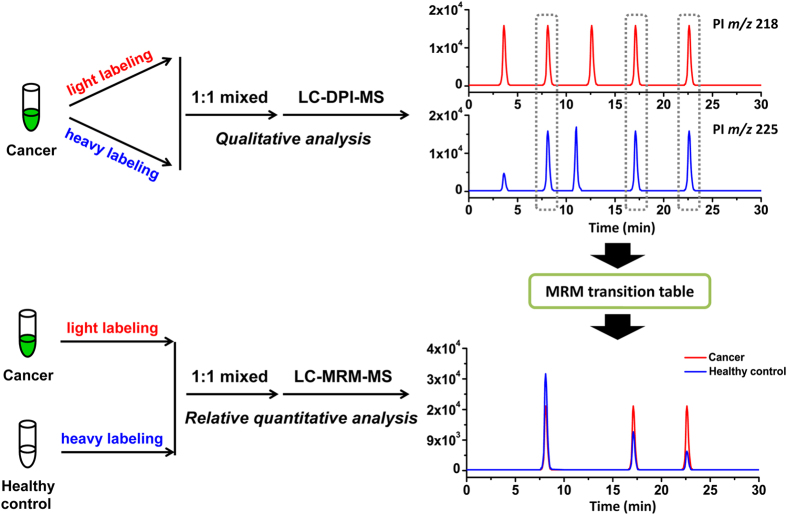
The schematic diagram of the principle of the developed method.

In our proposed strategy, the IL combined with LC-DPI-MS and LC-MRM-MS analysis was performed for the qualitative and relative quantitative analysis, respectively. The schematic diagram of the principle of this method was shown in Fig. 1.

### Optimization of TCEP and BQB conditions

The effect of the TCEP and BQB contents on reduction efficiencies of disulfide bonds and chemical labeling were investigated. Equal volumes of pooled urine samples from five cancer types and healthy control were mixed as the investigated samples. 5 compounds at *m/z* of 261, 377, 379, 401, 429 and retention times of 4.7, 16.5, 21.4, 27.9, and 33.6 min were extracted, and their peak areas were calculated to evaluate the TCEP and BQB effects.

Determination of thiols is complicated due to their occurrence in multiple forms, since their free sulfhydryl group is prone to oxidation^9^. TCEP has frequently been used as reducing agent and is considered as a suitable choice for low molecular weight disulfides^1^. The reducing effect of TCEP was investigated in the range of 10–500 nmol and the content of BQB was fixed at 100 nmol. As shown in Figure S1A, the peak areas of five thiol derivatives increased with the increase in TCEP concentration from 10 to 100 nmol. Whereas, the peak area dropped with further increase in TCEP concentration (>100 nmol) for the four analytes (*m/z* 377, 379, 401, and 429), and 200 nmol for the fifth (*m/z* 261), indicating that the efficiencies of chemical labeling may be suppressed by the excess of TCEP. Consequently, for identification of most of thiols in urine, 100 nmol of TCEP was used in the following experiments.

The amount of labeling reagent ranging from 5 to 200 nmol was further optimized. As shown in Figure S1B, the peak areas of thiol derivatives increased with the increase of BQB content from 5 to 20 nmol and finally reached a plateau when BQB contents exceeded 20 nmol. For more reliable quantitation of thiols in urine, 50 nmol of BQB was used in the following experiments.

### Qualitative analysis of thiols in human urine by IL-LC-DPI-MS

**Figure 2:**
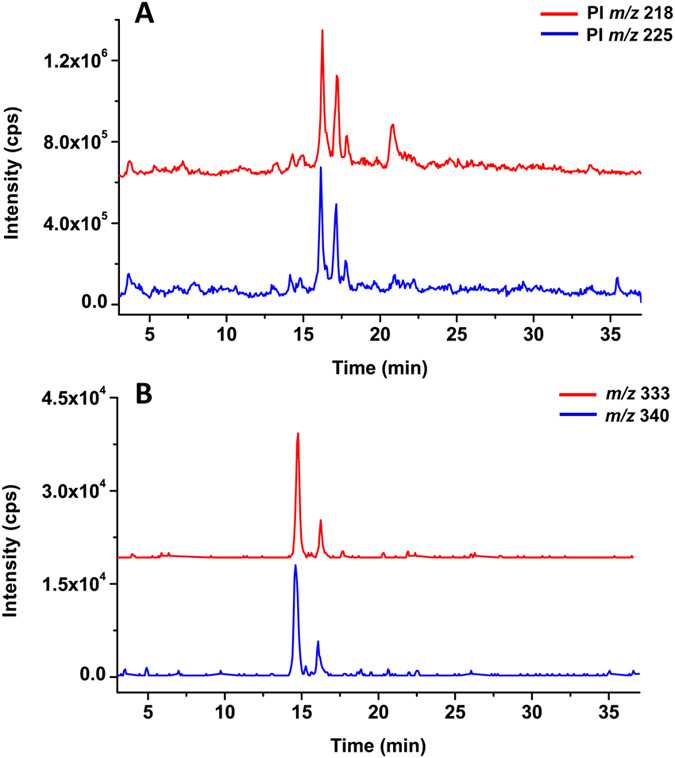
BQB and BQB-*d*_*7*_ labeled urine in nasopharyngeal cancer analyzed by IL-LC-DPI-MS. (**A**) Total ion chromatogram of DPI analysis; (**B**) Extracted ion chromatograms of *m/z* 333 and 340 from BQB and BQB-*d*_*7*_labeled ion chromatograms, respectively.

After optimization, we qualitatively analyzed the presence of thiols in human urine by IL-LC-DPI-MS method and pooled samples (n = 10) of each cancer type were prepared to minimize the variation between individuals. Figure 2A shows the total ion chromatograms of urine in nasopharyngeal cancer analyzed by IL-LC-DPI-MS. The two chromatograms derived from the BQB and BQB-*d*_*7*_ labeled urine samples displayed almost identical peak patterns. The other four cancer samples (i.e., esophagus cancer, gastric cancer, lymph cancer and lung cancer) analyzed by LC-DPI-MS were shown in Figure S2. Extracted peak-pair data from the two ion chromatograms according to a mass difference of 7 Da (i.e., M_BQB-*d7* labeled_ − M_BQB labeled_ = 7 Da) and only peak pairs with the same retention time and intensity were assigned to be the thiol candidates. Taking compound 7 and 8 as the examples (Fig. 2B), two peak pairs at retention times of 14.4 and 16.3 min were observed between the extracted ion chromatograms at *m/z* 333 and 340 from BQB and BQB-*d*_*7*_ labeled samples, respectively. Same peak intensities and retention times of those two peaks in two labeled chromatograms suggest that these two compounds were all thiol candidates. The structures of all the identified thiols were further elucidated by product-ion scan (MS/MS) and high resolution mass spectrometry (QTOF-MS) analysis.

In our previous research, a phenomenon was observed that if the compound containing n sulfhydryl groups (i.e., n = 2–6), all of the sulfhydryl groups could be labeled with BQB, and highest intensity peaks of these derivatives with n charge states (i.e., [M + n × BQB]^n+^) were observed among several of precursor ions with different charge states^22^. Similar peak pattern was observed in case of the BQB-*d*_*7*_ labeled compounds with *m/z* of ([M +  n × BQB-*d*_*7*_]^n+^). The mass shift of BQB and BQB-*d*_*7*_ labeled derivatives was always 7 Da no matter the compounds contain one or more sulfhydryl groups. To distinguish the numbers of sulfhydryl groups in thiol candidates, the charge number of the precursor ions from derivatives should be examined.

The charge numbers of thiol were further examined by QTOF-MS analysis and total 103 ion pairs were detected in all the 5 types of cancer urine samples (Table 1). These results were consistent with the results obtained from healthy urine in previous report^25^. Most of the detected thiols contain single charge, indicating that most of thiols only have one sulfhydryl group. The other 19 compounds could not be assigned the charge number by the QTOF data analysis software, which may be attributed to the low abundance of those thiols and the ion suppression effect of matrix interference.

Among the 103 identified thiols, 5 (No. 1–5) have been recognized as Cys, HCys, Nac, γ-GluCys, and GSH by the standards, and 12 (No. 6–17) were given the possible structures by the MS/MS and QTOF-MS information from our previous work^25^. In current research, it was found through standard comparison that the compound no. 6 named as cysteamine in previous reports, was not actually cysteamine(data not shown). Also, we found that the prospective molecular formulas (C_11_H_22_N_2_O_4_S) of compound 82 was identical to pantetheine, which have been found in *Arabidopsis thaliana* extracts^30^. Through comparison of retention times (Figure S3) and MS/MS data (Figure S4) with standards, compound 82 was identified to be pantetheine. The proposed structures of product ion derived from MS/MS spectra of pantetheine are also shown in the Figure S5. It is worth noting that the existence of pantetheine in urine is first reported by our developed method.

### Development of IL-LC-MRM-MS method

We further investigated the content changes of the 103 thiols in 5 types of cancer urine compared to healthy control by the IL-LC-DPI-MS method. The peak area ratios of each identified thiol from the cancer urines relative to healthy control were calculated by the forward and reverse labeling tests. However, results showed that only 64 (62%) of the 103 thiols could be calculated with the RSDs lower than 30% (data not shown). Among the other 38% of thiols not being quantified, which have lower signals or the signals were originally near the limit of quantifications (LOQs), were largely affected by the instrument signal fluctuations. So, the calculated RSDs were higher than 30% or their signals became lower than LOQs.

LC-MS operated in MRM mode has been widespread for targeted metabolite quantification^31^. The precursor ion and corresponding product ion of metabolite is monitored simultaneously in MRM mode, which improves the detection specificity and sensitivity by reducing the matrix interferences signals. In current study, we proposed an IL-LC-MRM-MS method to investigate the content changes of thiol between cancers and healthy controls. In this method, the product ion was fixed at *m/z* 218.1 and 225.1 for the BQB and BQB-*d*_*7*_ labeled compounds, respectively, and the precursor ions were acquired from the urine samples through the aforementioned non-targeted IL-LC-DPI-MS analysis. The MRM transitions of [M]^+^ → 218.1 and [M + 7]^+^ → 225.1 for BQB and BQB-*d*_*7*_ labeled thiols, respectively, were applied in the developed IL-LC-MRM-MS method.

**Figure 3:**
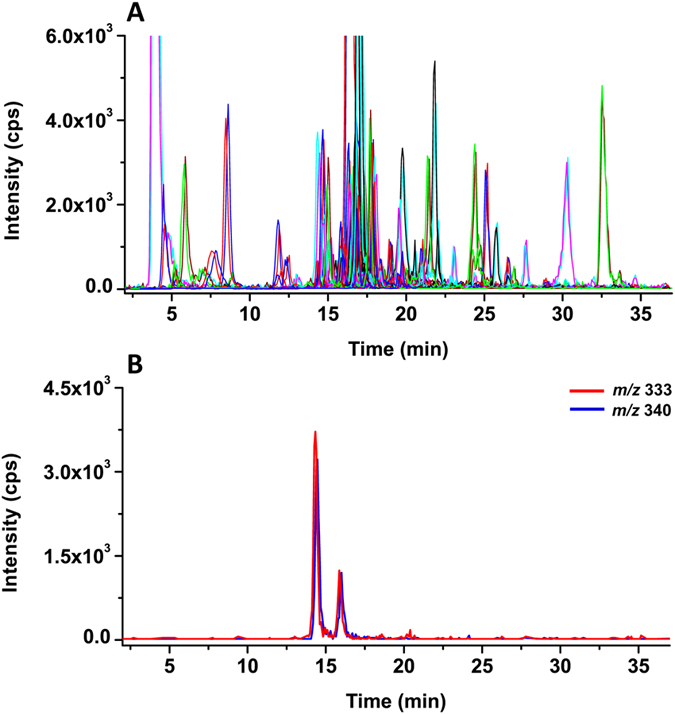
BQB and BQB-*d*_*7*_ labeled urine in nasopharyngeal cancer analyzed by IL-LC-MRM-MS. (**A**) Total ion chromatogram of MRM analysis; (**B**) Extracted ion chromatograms of *m/z* 333 and 340 from total ion chromatograms.

For comparison, the mixed BQB and BQB-*d*_*7*_ labeled nasopharyngeal cancer urine (1/1, v/v) was also examined by the IL-LC-MRM-MS method. The 206 ion transitions for the BQB and BQB-*d*_*7*_ labeled thiols are used in the MRMmethod. Figure 3A shows the extracted ion chromatograms of nasopharyngeal cancer urine analyzed by IL-LC-MRM-MS. In contrast to the DPI methods, all the MRM information was generated in single spectrum. Similar with DPI method, the peak intensities of *m/z* 333 and 340 from BQB and BQB-*d*_*7*_ labeled thiols at retention times of 14.4 and 16.3 min in the MRM mode were also same (Fig. 3B). We also compared the S/N of DPI and MRM methods. Result shows that the S/N significantly improved by 2.1–11.3 folds in MRM compared to DPI method. For example, the S/N of compounds 75 and 80 in the DPI analysis (Figure S6A,B) improved by 5.3 and 7.1 folds by MRM method, respectively (Figure S6C,D).

To evaluate the accuracy of the relative quantification obtained by our developed method, BQB and BQB-*d*_*7*_ labeled pooled urines were mixed at different volume ratios (1:10, 1:5, 1:2, 1:1, 2:1, 5:1, and 10:1) and the samples were analyzed by LC-MRM-MS in triplicate measurements. The peak area ratios of BQB/BQB-*d*_*7*_ labeled samples were calculated from sixty peak pairs with high intensities. The obtained average isotopic ratios were 0.1, 0.2, 0.5, 1.0, 2.1, 5.3, and 10.3 for the 1:10, 1:5, 1:2, 1:1, 2:1, 5:1, and 10:1 mixtures, respectively, with relative standard deviations (RSDs) being less than 9.0%. The correlation coefficient (R^2^) was 0.9998 and the slopes of linear regressions were approximately 1.00 (1.0291), which shows that the peak area ratios highly matched with the concentration ratios of the different isotope labeled analytes (Figure S7).

### Relative quantitative analysis of thiols in human urine by IL-LC-MRM-MS

We further investigated the content changes of the 103 thiols in 5 types of cancer urine samples (nasopharyngeal cancer, esophagus cancer, gastric cancer, lymph cancer, and lung cancer) compared to the healthy controls by our developed IL-LC-MRM-MS method. Before analysis, the creatinine in each pooled samples were quantified according to previously reported method^16^,^32^,^33^,^34^ (Table S1). The creatinine is a standard manner to normalize the concentrations of urine sample since the excretion of creatinine is rather constant over a longer time interval. The results of the measured peak areas ratios (cancer/healthy) are shown in Table S2. It is worth noting that, compared to the LC-DPI-MS method, the number of thiols being accurately quantified changed from 64 (62%) to 99 (96%) by the LC-MRM-MS method.

**Figure 4:**
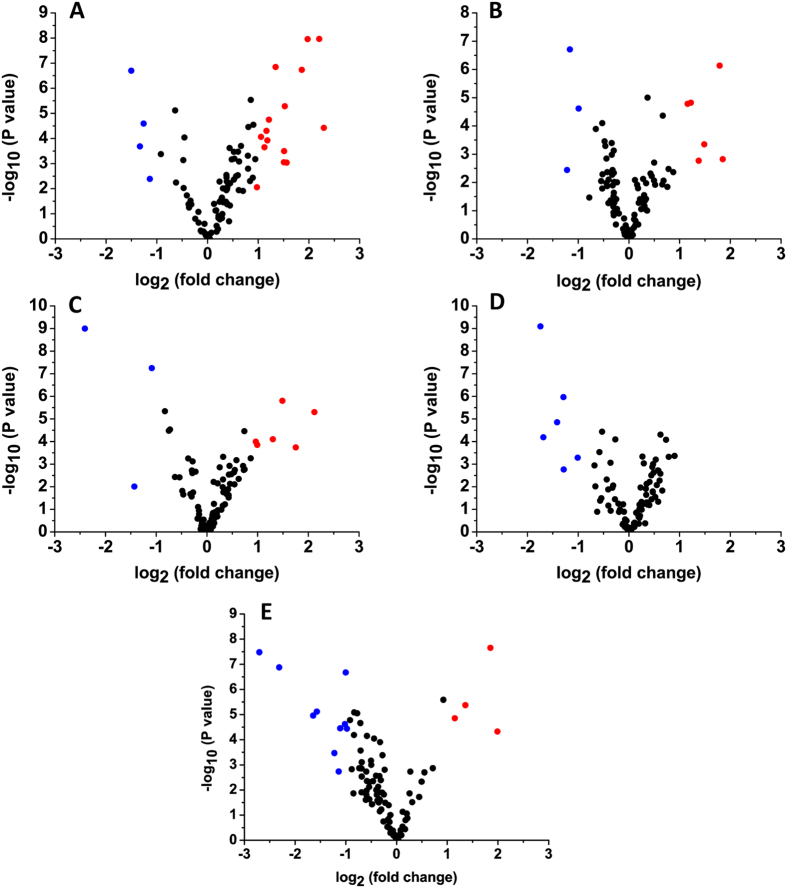
Volcano plots of thiol contents between cancers and healthy control. (**A**) nasopharyngeal cancer; (**B**) esophagus cancer; (**C**) gastric cancer; (**D**) lymph cancer; (**E**) lung cancer; *p* = 0.01; 2.0-fold changes.

The unpaired student’s *t*-test was performed to examine the statistical significance of fold changes from six independently biological experiments (three from forward labeling and the other three from reverse labeling). The result of statistical test including *p*-values and 95% confidence interval estimates are shown in Table S3. The fold change of increased or decreased more than 2.0 and the *p*-values less than 0.01 were considered as a statistically significant difference. Figure 4 shows a volcano plot, where the –log_10_ (*p* value) was plotted against its corresponding log_2_ (fold change of cancer/healthy control). The blue plots represent the significantly decreased and the red plots represent the significantly increased thiols compared to healthy controls.

As shown in Table 2, amongst the decreased thiols, compound 32 decreased more than 2.0 folds in all types of cancers, which may be employed as potential indicator for the screening of cancers. Similarly, compound 77 decreased more than 2.0 folds in 3 types of cancers (nasopharyngeal cancer, lymph cancer, and lung cancer), whereas, it increased more than 2.0 folds in the gastric cancer (Table 3). Compounds 6, 12, 33, and 82 (pantetheine) were also found more than 2.0 folds decrease in nasopharyngeal and lymph cancer, lymph and lung cancer, nasopharyngeal and gastric cancer, and esophagus and lung cancer, respectively. Interestingly, compounds 12 also increased more than 2.0 folds in the esophagus cancer (Table 3).

For the increased thiols, as shown in the Table 3, compound 40 was found more than 2.0 folds increase in 3 types of cancers (nasopharyngeal cancer, esophagus cancer and gastric cancer). Compound 41 increased 2.0 foldsin nasopharyngeal and esophagus cancers. Both compounds 18 and 56 increased 2.0 folds in esophagus and gastric cancers, but compounds 18 decreased more than 2.0 folds in lung cancer (Table 2). All 5 types of cancers, including nasopharyngeal cancer, esophagus cancer, gastric cancer, and lung cancer, have their own characteristic thiols with increased level, except lymph cancer. It is worth noting that the two known thiols of HCys and γ-GluCys increased 2.0 folds in nasopharyngeal cancer and gastric cancer, respectively. The result of HCys was consistent to the previous reports on plasma sample analysis from breast, colorectal, and cervical cancer^2^,^3^,^4^,^6^. However, there was no study reported so far about the content changes of thiol in urine of cancer. Thus, our study presents the first report for the increased level of HCys and γ-GluCys in urine of nasopharyngeal cancer and gastric cancer, respectively.

For further elucidation of significantly increased and decreased thiols in cancer (Tables 2 and 3), 3 compounds (pantetheine, HCys and γ-GluCys) were successfully identified by comparing to the standards; and 2 compounds (compound 11 and 12) were given the possible structures by MS/MS and QTOF-MS information. However, most of the compounds could not be identified. We could not found their structures in the HMDB and METLIN database by the prospective formulas or molecular weight information, suggesting that they may not been previously found in biological samples. To give reference in the following study, we could found prospective structures in the ChemSpider database, which is a free chemical structure database providing fast structure search access to over 35 million structures from hundreds of data sources.

In order to reach a reliable result, we took all types of 50 cancer urines as “cancer samples”, also collected 30 healthy urines as “healthy control”, then, the pooled samples of cancer and healthy control were labeled with BQB and BQB-*d*_*7*_ to form the forward and reverse labeling samples to access the content changes of thiol in mixed cancers compared to healthy control (Table S4). As shown in Table S4, compound 32 was decreased in the mixed cancers, the average peak area ratios (cancer/healthy control) was about 0.6, similar to the results from individual types of cancer (Table S2, from 0.3 to 0.6). Some other thiols, which were significantly increased or decreased in their corresponding cancers compared to healthy controls, have not been found changes through mixing all types of cancer as one sample pool, i.e., compound 4 (γ-GluCys) in gastric cancer and compound 102 in lung cancer. So, future research should be focused on the individual types of cancer and more numbers of sample would be analyzed to verify our findings.

## Discussion

Biothiol imbalances in biological samples are associated with different kinds of disease, such as cardiovascular disease, neurodegenerative disease, cancer, kidney dysfunction, and diabetes mellitus^1^. Based on these biochemical findings, there is a growing interest in identifying biomarkers for diseases in which thiols are involved. For example, HCys is considered as a biomarker in the cardiovascular disease^7^, and the disease of cystinuria can also be characterized by excessive urinary excretion of Cys^8^.

Here, we developed a novel method for the comprehensive analysis of thiols in 5 types of cancer urine. In this method, the IL-DPI-LC-MS was firstly applied for non-targeted profiling of thiols in 5 cancer urines. The DPI method can significantly improve the identification accuracy by generating two individual ion chromatograms corresponding to BQB and BQB-*d*_*7*_ labeled urines. Using this strategy, 103 thiol candidates were discovered in all the cancer urines and 6 thiol candidates in urines were confirmed as Cys, HCys, Nac, γ-GluCys, GSH and pantetheine by standards. In these identified thiol compounds, pantetheine has been firstly discovered in human urine, which extends the diversity of the thiol metabolites present in human urine. The pantetheine is considered as an intermediate in the production of coenzyme A in mammalian liver by preparation and purification of enzymes *in vitro*^35^. However, through comparative genomics, pantetheine was not found in coenzyme A biosynthesis pathway in the body^36^. However, it is unclear whether the pantetheine detected in urine is the metabolic product of 4'-phosphopantetheine, dephosphocoenzyme A, or coenzyme A, all of which have pantetheine structure moiety and are considered as cofactors in coenzyme A biosynthesis by the body, or an intermediate in other biological pathway. Thus, it is essential to investigate this compound in human cell extracts to elucidate its existence and biological pathway in our future work.

Then, the IL-LC-MRM-MS method was firstly developed and applied to compare content changes in 5 types of cancer and healthy controls. Compared to DPI method, the quantification sensitivity of MRM method improved by 2.1–11.3 folds. Then, the number of compounds, which could be accurately quantified, changed from 64 (62%) to 99 (96%). We found that different content changes of thiols are associated with different types of cancers. Every cancer has their own characteristic thiols which significantly increased or decreased compared to healthy controls. The phenomenon may be due to the heterogeneity of different cancers. The HCys and γ-GluCys were firstly reported more than 2.0 folds increase in the urine of nasopharyngeal cancer and gastric cancer, respectively, then, the two thiols could be considered as potential biomarker for the nasopharyngeal and gastric cancers. The pantetheine were found more than 2.0 folds decrease in both esophagus and lung cancer urines. In addition, compounds 32 decreased more than 2.0 folds in urines of all the examined types of cancers, which may be employed as potential indicator for the screening of cancers. However, most of the compounds that showed significant changes could not be identified. So, further study should focus on the identification of thiols and provide an insight into the better use of urinary thiols as biomarkers for cancers. Taken together, the IL-LC-DPIS-MS method combined with IL-LC-MRM-MS method demonstrated to be a promising strategy for the identification and quantification of compounds with identical groups in metabolomics study.

## Methods

### Reagents

Cysteine (Cys), homocysteine (HCys), *N*-acetyl-cysteine (Nac), γ-glutamylcysteine (γ-GluCys), glutathione (GSH), pantetheine, and glycine were purchased from Sigma (St. Louis, MO, USA). Chromatographic grade methanol was purchased from TEDIA Co. Inc. (Ohio, USA). Formic acid and ethylenediaminetetraacetic acid (EDTA) were purchased from Sinopharm Chemical Reagent Co., Ltd. (Shanghai, China). All other solvents and chemicals used were of analytical grade. The water used throughout the study was purified by a Milli-Q apparatus (Millipore, Bedford, MA). Stock standard solutions of Cys, HCys, Nac, γ-GluCys, GSH, and pantetheine were prepared in 1.0 mmol/L ethylenediaminetetraacetic acid (EDTA) solution containing 0.05% formic acid at a concentration of 1.0 mmol/L.

### Urine sample collection and preparation

The 5 types of cancer (nasopharyngeal cancer, esophagus cancer, gastric cancer, lymph cancer, and lung cancer) and healthy control were collected from Hubei Cancer Hospital, China. The 10 samples of first morning urine of every types of cancer and healthy controls were collected (5 males and 5 females; 60 ± 5 years old). All the patients were diagnosed with cancer for the first time and had not been given any treatment at the time point of urine samples collection. Healthy controls were selected based on medical history and physical examination. Written informed consent was obtained from the study subjects, and an approval was granted by the Hubei Cancer Hospital Ethics Committee and met the declaration of Helsinki. All the experiments were performed in accordance with Hubei Cancer Hospital Ethics Committee’s guidelines and regulations.

The urine samples were pretreated according to previously described method^16^. Briefly, 200 μL of each urine sample was added to a prepared screw-cap vial (1.5 ml) containing 18 μL of EDTA (10 mmol/L) and 2 μL of formic acid. Six pooled samples of five cancers and healthy control were prepared by taking equal volume of their 10 urine samples. Then 100 μL of pooled sample was treated with 100 nmol of tris (2-carboxyethyl) phosphine hydrochloride (TCEP, 10 mmol/L, 10 μL) under 45 °C for 60 min.

### Principle of the strategy

Firstly, each type of cancer urine was subjected to IL-LC-DPI-MS method for the non-targeted profiling of thiols. Equal volume of sample was labeled with BQB and BQB-*d*_*7*_, respectively. Then the light and heavy labeled samples were mixed and analyzed by LC-DPI-MS. The LC-DPI-MS method generated two individual ion chromatograms corresponding to the precursor ion of BQB and BQB-*d*_*7*_ labeled thiols, respectively. Peak-pair data were extracted from the two ion chromatograms according to a characteristic mass difference and only peak pairs with the same retention time and intensity were assigned as thiol candidates. Secondly, the targeted relative quantification of the thiols between the cancer and healthy control was investigated by the IL-LC-MRM-MS method. All the MRM transitions of [M]^+^ → 218.1 and [M + 7]^+^ → 225.1 for BQB and BQB-*d*_*7*_ labeled thiols, respectively, were quantified in the LC-MRM-MS, in which [M]^+^ and [M + 7]^+^ were generated from DPI results in the urine samples.

### Qualitative analysis of thiols in urine

The qualitative analysis of thiols in cancer urines by IL-LC-DPI-MS method was performed according to our previous work^25^. Briefly, 50 nmol of BQB or BQB-*d*_*7*_ (1 mm, 50 μL) was added to a 1.5-mL tube and dried under nitrogen gas. Subsequently, 100 μL of Gly-HCl buffer solution (5.0 mmol/L, pH 3.5) and 10 μL of urine were added. The mixture was incubated at 60 °C for 60 min with shaking at 1,500 rpm. Then equal volume of BQB and BQB-*d*_*7*_ labeled sample solutions were mixed and 50 μL of the solution was subjected to LC-DPI-MS analysis.

### Relative quantitative analysis of thiols in urine

The cancer and healthy control urine samples were labeled with BQB and BQB-*d*_*7*_ to form the forward and reverse labeling sample solutions to invest the content changes of thiol in cancers compared to healthy control. In the forward labeling, the cancer sample was labeled with BQB and the healthy control sample was labeled by BQB-*d*_*7*_. In the reversed labeling, the cancer sample was labeled with BQB-*d*_*7*_ and the healthy control sample was labeled by BQB. Then the two labeled samples were mixed (1:1, v/v) and analyzed by LC-MRM-MS. Triplicate measurements were performed in each labeling strategy.

### LC-DPI-MS analysis

Analysis of sample was performed on the LC-ESI-MS/MS system consisting of an AB 3200 QTRAP mass spectrometer (Applied Biosystems, Foster City, CA, USA) with an electrospray ionization source (Turbo Ionspray) and a Shimadzu LC-20AD HPLC (Tokyo, Japan) with two LC-20AD pumps, a SIL-20A auto sampler, a CTO-20AC thermostated column compartment and a DGU-20A3 degasser. Data acquisition and processing were performed using AB SCIEX Analyst 1.5 Software (Applied Biosystems, Foster City, CA, USA). The HPLC separation was performed on a Shimadzu VP-ODS column (150 mm × 2.0 mm i.d., 5 μm, Tokyo, Japan) with a flow rate of 0.2 mL/min at 30 °C. Formic acid in water (0.1%, v/v, solvent A) and methanol (solvent B) were employed as mobile phase. A gradient of 0–5 min 5% B, 5–35 min 5% to 60% B, 35–40 min 60% to 5% B, and 40–55 min 5% B was used.

The DPI method consists of two PI (*m/z* 218 and 225) in the mass range of *m/z* 200–600. DPI was carried out under positive ion mode. IsoSpray voltage was set at 5.2 kV and vaporizer temperature was set at 550 °C. The mass spectrometer was operated with gas settings of 40 psi for nebulizer gas, 30 psi for curtain gas, and 60 psi for collision gas. Scan time per cycle was 2.0 s with a pause of 5.0 ms for each scan. Resolution of Q1 and Q3 was set to “low” and “unit”, respectively. Declustering potential, entrance potential, cell entrance potential, collision energy and cell exit potential were set at 45 V, 7 V, 15 V, 38 V and 3 V, respectively.

For structural identification (MS/MS analysis), IDA (Information Dependent Acquisition) mode was performed under positive ion mode. The criteria were set as that EPI was triggered when signals of the pre-selected compounds by PI exceeding 1000 counts/s at their retention times. The mass tolerance was set to 250 mDa, and retention time tolerance was set to 60 s.

### LC-MRM-MS analysis

The relative quantification of thiols between the cancer and healthy urines was performed by LC-MRM-MS in positive mode. The transitions of [M]^+^ → 218.1 and [M + 7]^+^ → 225.1 for BQB and BQB-*d*_*7*_ labeled thiols, respectively, were detected as MRM ion pairs. All the precursor ions ([M]^+^ and [M + 7]^+^) for the MRM quantification were derived from the DPI method in the urine samples. The LC condition and the mass spectrometer parameters for quantification of thiols were identical with the aforementioned LC-DPI-MS method.

### LC-QTOF-MS analysis

High resolution mass spectrometry experiments was performed on the LC-QTOF-MS system consisting of a MicrOTOF-Q orthogonal-accelerated TOF mass spectrometer (Bruker Daltonics, Bremen, Germany) with an ESI source (Turbo Ionspray) and a Shimadzu LC-20AB binary pump HPLC (Tokyo, Japan), a SIL-20AC auto sampler, and a DGU-20A_3_ degasser. Data acquisition and processing were performed using Bruker Daltonics Control 3.4 and Bruker Daltonics Data analysis 4.0 software. The HPLC separation column and mobile phase gradient were same as that of LC-DPI-MS method.

The mixture of BQB and BQB-*d*_*7*_ labeled samples (1/1, v/v) were detected under positive ion mode. The optimized ESI parameters were as follows: capillary voltage −4.5 kV; dry gas 5.0 L/min; dry temperature 180 °C; funnel 1 RF 200.0 Vpp; funnel 2 RF 200.0 Vpp; ISCID energy 0.0 eV; hexapole RF 200.0 Vpp; pre pulse storage 12.0 μs. Spectra were acquired by summarizing 5000 single spectra. Full scan mode was used.

The prospective molecular formulas of BQB-thiol derivatives were generated based on the accurate mass and isotope patterns of elemental composition using Bruker Daltonics Data analysis 4.0 software. A mass tolerance of 5.0 mDa was set and a maximum elemental composition of C = 50, H = 100, N = 50, O = 50, S = 10, P = 10, and Cl = 10 was used. The molecular formulas of thiols were obtained by subtracting the molecular formula of BQB (C_12_H_10_NO). The molecular formulas obtained by TOF was further searched in the database of METLIN (http://metlin.scripps.edu/index.php) and HMDB (http://www.hmdb.ca/metabolites) for putative identification.

## Additional Information

**How to cite this article**: Liu, P. *et al.* Determination of thiol metabolites in human urine by stable isotope labeling in combination with pseudo-targeted mass spectrometry analysis. *Sci. Rep.* **6**, 21433; doi: 10.1038/srep21433 (2016).

## Supplementary Material

Supplementary Information

## Figures and Tables

**Table 1:** The measured *m/z* of BQB and BQB-*d*_*7*_ labeled thiols in human cancer urines and their prospective molecular formulas obtained by QTOF analysis.

No.	T/min	Charge number	BQB labeled (*m/z*)	BQB-*d*_*7*_labeled (*m/z*)	Error (mDa)	Prospective formulas	Compounds name
1	3.7	1	305.1001	312.1412	4.1	C_3_H_7_NO_2_S(121.0197)	Cysteine
2	4.4	1	319.1163	326.1503	4.7	C_4_H_9_NO_2_S(135.0354)	Homocysteine
3	16.1	1	347.1073	354.1510	0.7	C_5_H_9_NO_3_S(163.0303)	*N*-acetylcysteine
4	7.9	1	434.1407	441.1831	2.1	C_8_H_14_N_2_O_5_S(250.0623)	γ-Glutamylcysteine
5	8.6	1	491.1661	498.2106	6.1	C_10_H_17_N_3_O_6_S(307.0838)	Glutathione
6	4.7	1	261.1031	268.1365	−3.1	C_2_H_7_NS (77.0299)	
7	14.4	1	333.0918	340.1327	0.9	C_4_H_7_NO_3_S(149.0147)	Formylcysteine
8	16.3	1	333.0888	340.1223	−2.1	C_4_H_7_NO_3_S(149.0147)	(2-Mercaptoacetyl)glycine
9	18.6	1	361.1240	368.1657	1.8	C_6_H_11_NO_3_S(177.0460)	(*Z*)-(2-(mercaptomethyl)-3-methoxyallyl)carbamic acid
10	23.6	1	375.1006	382.1400	−0.9	C_6_H_9_NO_4_S(191.0252)	(*Z*)-2-(((*Z*)-1-hydroxy-3-methoxyprop-1-en-1-yl)imino)-2-mercaptoacetic acid
11	17.9	1	377.1197	384.1620	2.6	C_6_H_11_NO_4_S (193.0409)	2-((2-Hydroxyethyl)amino)-3-mercapto-4-oxobutanoic acid
12	23.1	1	382.1552	389.1876	−3.7	C_9_H_14_N_2_OS(198.0827)	5-Amino-1-(5-mercapto-2*H*-pyrrol-3-yl)pentan-2-one
13	26.6	1	403.1467	410.1815	−1.3	C_12_H_13_NOS(219.0718)	7-(Mercaptomethyl)-1,2-dihydronaphthalene-2-carboxamide
14	17.1	1	405.1165	412.1595	4.5	C_7_H_11_NO_5_S(221.0358)	(*E*)-3-(4,4-dihydroxybut-2-enamido)-2-mercaptopropanoic acid
15	15.3	1	407.1213	414.1589	3.5	C_9_H_9_N_3_O_2_S(223.0415)	Methyl 4-(4-(mercaptomethyl)-1,3,5-triazin-2-yl)but-3-ynoate
16	16.4	1	407.1311	414.1718	3.4	C_7_H_13_NO_5_S(223.0514)	4-((1-Carboxy-2-mercaptoethyl)amino)-2-hydroxybutanoic acid
17	16.6	1	421.1161	428.1619	0.4	C_10_H_11_N_3_S_2_(237.0394)	5-(2-(Pyrazin-2-ylamino)ethyl)thiophene-3-thiol
18	15.8	1	298.0255	305.0691	−4.8	H_3_O_3_PS(113.9541)	
19	16.8	1	303.1160	310.1579	−0.7	C_4_H_9_NOS(119.0405)	
20	14.3	1	308.0787	315.1216	0.8	C_3_H_8_OS_2_(124.0017)	
21	11.8		348.0994	355.1364	−2.4	C_4_H_8_N_2_O_3_S(164.0256)	
22	17.8	1	361.1206	368.1654	−1.6	C_6_H_11_NO_3_S(177.0460)	
23	7.0	1	362.1178	369.1542	0.3	C_5_H_10_N_2_O_3_S(178.0412)	
24	14.6		363.1036	370.1438	2.1	C_5_H_9_NO_4_S(179.02520)	
25	16.1	1	365.1190	372.1608	1.9	C_5_H_11_NO_4_S (181.0409)	
26	28.7		366.1242	373.1602	−3.4	C_8_H_10_N_2_OS(182.0514)	
27	16.4	1	371.1142	378.1288	−3.6	C_6_H_9_N_3_O_2_S(187.0415)	
28	17.1	1	371.1109	378.1513	1.4	C_6_H_10_N_3_PS(187.0333)	
29	21.8	1	376.1171	383.1480	1.8	C_6_H_12_N_2_OS_2_(192.0319)	
30	25.4	1	376.1557	383.1950	−2.6	C_8_H_16_O_3_S(192.0820)	
31	16.5	1	377.1133	384.1578	−3.8	C_6_H_11_NO_4_S(193.0409)	
32	21.4		379.0840	386.1240			
33	6.3		380.1217	387.1628	−2.1	C_10_H_13_PS(196.0476)	
34	17.5	1	389.1233	396.1612	1.5	C_5_H_11_N_5_S_2_(205.0456)	
35	24.5	1	389.1492	396.1861	2.2	C_7_H_15_N_3_S_2_(205.-0707)	
36	16.1	1	391.1024	398.1443	−1.0	C_10_H_10_NPS(207.0272)	
37	17.0	1	391.1361	398.1718	3.3	C_7_H_13_NO_4_S(207.0565)	
38	18.4	1	391.1277	398.1699	4.8	C_9_H_9_N_3_OS(207.0466)	
39	21.1	1	391.1374	398.1502	1.6	C_6_H_14_N_3_OPS(207.0595)	
40	27.9	1	401.1503	408.1923	−3.2	C_9_H_15_NO_3_S(217.0773)	
41	28.5	1	403.1642	410.2093	−5.0	C_9_H_17_NO_3_S(219.0929)	
42	29.1	1	403.1390	410.1752	1.5	C_6_H_13_N_5_S_2_(219.0612)	
43	16.9	1	407.1388	414.1671	−0.1	C_6_H_13_N_3_O_4_S(223.0627)	
44	17.2	1	407.1159	414.1553	−1.9	C_9_H_9_N_3_O_2_S(223.0415)	
45	23.3	1	412.1200	419.1600	−1.9	C_10_H_12_O_4_S(228.0456)	
46	17.2	1	415.1042	422.1455	0.8	C_12_H_10_NPS(231.0272)	
47	16.7	1	416.1312	423.1739	−0.8	C_13_H_12_O_2_S(232.0558)	
48	30.2	1	417.1522	424.1902	−0.9	C_7_H_15_N_5_S_2_(233.0769)	
49	15.8	1	419.1342	426.1746	−4.7	C_7_H_13_N_3_O_4_S(235.0627)	
50	18.9	1	419.1310	426.1728	−1.4	C_6_H_13_N_5_OS_2_(235.0562)	
51	27.6	1	419.1650	426.1935	0.9	C_9_H_17_NO_4_S(235.0878)	
52	5.8	1	420.1267	427.1708	−0.3	C_12_H_12_O_3_S(236.0507)	
53	11.9	1	423.1218	430.1634	−0.8	C_7_H_13_NO_6_S(239.0464)	
54	12.7	1	423.1233	430.1645	−0.6	C_8_H_9_N_5_O_2_S(239.0477)	
55	17.1	1	423.1293	430.1643	−0.3	C_11_H_14_NOPS(239.0534)	
56	26.5	1	423.1417	430.1810	3.8	C_11_H_13_NO_3_S(239.0616)	
57	18.1	1	428.1322	435.1752	−0.5	C_7_H_12_N_6_S_2_ (244.0565)	
58	17.9	1	429.1350	436.1790	0.5	C_7_H_11_N_5_O_3_S(245.0583)	
59	21.1	1	429.1317	436.1666	1.0	C_10_H_15_NO_2_S_2_(245.0544)	
60	33.6	1	429.1814	436.2246	−0.7	C_7_H_15_N_7_OS(245.1059)	
61	19.8	1	432.1399	439.1652	−2.3	C_17_H_12_S(248.0660)	
62	16.5	1	433.1200	440.1603	1.0	C_8_H_15_N_3_S_3_(249.0428)	
63	17.2	1	433.1152	440.1564	−0.5	C_11_H_11_N_3_S_2_(249.0394)	
64	19.1	1	433.1120	440.1534	0.3	C_6_H_11_N_5_O_2_S_2_(249.0354)	
65	20.7	1	433.1436	440.1740	−1.1	C_10_H_11_N_5_OS(249.0684)	
66	22.3	1	433.1070	440.1501	−0.8	C_9_H_15_NOS_3_(249.0316)	
67	16.4	1	434.1407	441.1831	2.1	C_8_H_14_N_2_O_5_S(250.0623)	
68	16.5	1	435.1292	442.1724	−0.4	C_12_H_14_NOPS(251.0534)	
69	17.3	1	435.1338	442.1759	0.8	C_9_H_18_NOPS_2_(251.0567)	
70	19.2	1	437.1024	444.1378	−0.8	C_8_H_7_N_5_O_3_S(253.0270)	
71	21.4	1	444.1100	451.1489	−2.6	C_11_H_16_OS_3_(260.0363)	
72	33.8		447.1972	454.2188	−1.2	C_10_H_22_N_3_OPS (263.1221)	
73	16.2		448.1831	455.2268	0.7	C_10_H_21_N_2_O_2_PS(264.1061)	
74	19.2	1	449.1856	456.2307	−0.3	C_9_H_19_N_3_O_4_S(265.1096)	
75	14.2	1	450.1355	457.1794	0.7	C_9_H_10_N_6_O_2_S(266.0586)	
76	36.6	1	453.1857	460.2025	0.9	C_13_H_19_NO_3_S(269.1086)	
77	27.9	1	455.1477	462.2043	1.4	C_12_H_17_NO_2_S_2_(271.0701)	
78	5.90		459.1350	466.1824	1.2	C_9_H_13_N_3_O_5_S(275.0576)	
79	24.5	1	459.1570	466.2008	0.5	C_15_H_17_NS_2_(275.0802)	
80	26.0	1	461.1696	468.2122	0.2	C_10_H_21_N_3_P_2_S(277.0931)	
81	16.7	1	462.1415	469.1883	−0.7	C_12_H_14_N_4_S_2_(278.0660)	
82	20.9	1	462.2053	469.2489	−1.0	C_11_H_22_N_2_O_4_S(278.1300)	Pantetheine
83	19.8	1	472.1325	479.1699	1.8	C_7_H_17_N_2_O_6_PS(288.0545)	
84	7.13	1	477.1522	484.1836	−0.9	C_11_H_21_NO_2_P_2_S(293.0768)	
85	15.0		478.1374	485.1796	1.6	C_11_H_18_O_5_S_2_(294.0596)	
86	31.1		482.1559	489.1942	−2.0	C_6_H_18_N_8_S_3_(298.0817)	
87	16.1	1	490.1426	497.1854	−1.1	C_14_H_14_N_2_O_4_S(306.0674)	
88	17.2	1	490.1413	497.1824	3.0	C_6_H_6_N_14_S(306.0621)	
89	18.2	1	490.1382	497.1832	−0.1	C_6_H_6_N_14_S(306.0621)	
90	11.2	1	491.1640	498.2061	−0.8	C_8_H_17_N_7_O_2_S_2_(307.0885)	
91	18.6	1	506.1770	513.2228	−0.5	C_14_H_27_PS_3_(322.1012)	
92	21.2	1	506.1995	513.2462	2.5	C_13_H_26_N_2_OS_3_(322.1207)	
93	27.1		528.1679	535.1966	−0.1	C_19_H_22_P_2_S(344.0917)	
94	25.5		529.1958	536.2203	0.8	C_7_H_23_N_9_OS_3_(345.1188)	
95	17.3	1	533.1896	540.2346	−0.9	C_13_H_25_N_3_O_2_P_2_S(349.1143)	
96	15.9		544.1676	551.2043	−0.4	C_15_H_25_N_2_PS_3_(360.0917)	
97	14.7		546.1883	553.2194	−1.5	C_18_H_24_N_2_P_2_S(362.1135)	
98	14.0		549.2298	556.2612	−0.3	C_14_H_28_N_3_O_4_PS (365.1538)	
99	16.9		551.2046	558.2368	0.8	C_18_H_25_NO_3_S_2_(367.1276)	
100	18.0	1	563.2074	570.2559	−0.9	C_9_H_21_N_11_O_2_S_2_(379.1321)	
101	18.4		577.2231	584.2479	−1.0	C_17_H_33_NOP_2_S_2_(393.1479)	
102	20.1		577.2195	584.2909	0.0	C_20_H_27_NO_3_S_2_(393.1432)	
103	16.3		588.2067	595.2843	−2.3	C_18_H_28_O_6_S_2_(404.1327)	

**Table 2:** The thiols that decreased more than 2.0 folds in different types of cancer compared to healthy controls.

Cancer	NO.						NO.
	Commom compounds						Individual compounds
Nasopharyngeal cancer	32^c^	77	6^c^		33		
Esophagus cancer	32					82 (pantetheine)^a^	
Gastric cancer	32				33		
Lymph cancer	32	77	6	12^b^			45^c^
Lung cancer	32	77		12		82 (pantetheine)	18, 20, 28, 46, 76^c^, 102

^a^Identified by their respective standards.

^b^Given possible structures by the MS/MS and QTOF-MS information.

^c^Found possible structures on the ChemSpider database by their prospective formulas.

**Table 3:** The thiols that increased more than 2.0 folds in different types of cancer compared to healthy controls.

Cancer	NO.				NO.
	Commom compounds				Individual compounds
Nasopharyngeal cancer	40^c^	41^c^			2 (HCys)^a^, 11^b^, 23, 31^c^, 38^c^, 39, 52^c^, 53^c^, 65^c^, 67^c^, 75, 80, 100, 102
Esophagus cancer	40	41	18	56^c^	12^b^, 49
Gastric cancer	40		18	56	4 (γ-GluCys)^a^, 37^c^, 77
Lymph cancer					
Lung cancer					51^c^

^a^Identified by their respective standards.

^b^Given possible structures by the MS/MS and QTOF-MS information.

^c^Found possible structures on the ChemSpider database by their prospective formulas.
