# Supplementary material for: Determination of thiol metabolites in human urine by stable isotope labeling in combination with pseudo-targeted mass spectrometry analysis
Source: Sci Rep. 2016 Feb 18;6:21433. doi: 10.1038/srep21433 (PMC4757830; doi:10.1038/srep21433)

**Supporting Information**

**for**

**Determination of thiol metabolites in human urine by stable isotope labeling in combination with pseudo-targeted mass spectrometry analysis**

Ping Liu,^1,†^ Chu-Bo Qi,^1,2,†^ Quan-Fei Zhu,^1^ Bi-Feng Yuan,^1^ Yu-Qi Feng^1^,*

^1^ Key Laboratory of Analytical Chemistry for Biology and Medicine (Ministry of Education), Department of Chemistry, Wuhan University, Wuhan 430072, P.R. China

^2^ Department of Pathology, Hubei Cancer Hospital, Wuhan, Hubei 430079, P.R. China

^†^ These authors contributed equally to this work.

***To whom correspondence should be addressed. Tel.: +86-27-68755595; fax: +86-27-68755595. E-mail address: [yqfeng@whu.edu.cn](mailto:yqfeng@whu.edu.cn).

Table S1. The measured concentration of creatinine in each pooled urine samples.

|  | Concentration (mM, n = 3) |
| --- | --- |
| Nasopharyngeal cancer | 8.6 ± 0.4 |
| Esophagus cancer | 9.8 ± 0.8 |
| Gastric cancer | 12.9 ± 0.3 |
| Lymph cancer | 13.1 ± 0.7 |
| Lung cancer | 9.3 ± 0.3 |
| Healthy control | 11.1± 0.6 |

Table S2. List of the measured peak area ratios (cancer/healthy control) of 99 thiols from 5 cancer urines by IL-LC-MRM-MS method.

| NO. | Time  (min) | *m/z* | **Nasopharyngeal cancer** | | **Esophagus cancer** | | **Gastric cancer** | | **Lymph cancer** | | **Lung cancer** | |
| --- | --- | --- | --- | --- | --- | --- | --- | --- | --- | --- | --- | --- |
|  |  |  | Forward | Reverse | Forward | Reverse | Forward | Forward | Forward | Reverse | Forward | Reverse |
| 1 | 3.7 | 305.1 | 1.5 ± 0.1 | 1.6 ± 0.1 | 0.7 ± 0.0 | 0.8 ± 0.1 | 1.4 ± 0.2 | 1.2 ± 0.0 | 1.2 ± 0.3 | 1.3 ± 0.0 | 0.8 ± 0.1 | 0.8 ± 0.1 |
| 2 | 4.4 | 319.1 | 2.1 ± 0.1 | 2.4 ± 0.1 | 1.4 ± 0.0 | 1.7 ± 0.0 | 0.4 ± 0.0 | 0.5 ± 0.0 | 1.4 ± 0.0 | 1.5 ± 0.2 | 0.8 ± 0.0 | 0.8 ± 0.1 |
| 3 | 16.1 | 347.1 | 1.1 ± 0.0 | 1.1 ± 0.1 | 1.1 ± 0.0 | 1.1 ± 0.0 | 1.1 ± 0.0 | 1.1 ± 0.0 | 1.0 ± 0.0 | 1.0 ± 0.0 | 0.7 ± 0.0 | 0.7 ± 0.1 |
| 4 | 7.9 | 434.1 | 0.8 ± 0.1 | 0.7 ± 0.1 | 0.8 ± 0.0 | 0.9 ± 0.1 | 2.4 ± 0.4 | 2.5 ± 0.2 | 1.0 ± 0.3 | 0.9 ± 0.1 | 0.7 ± 0.0 | 0.7 ± 0.0 |
| 5 | 8.6 | 491.2 | 0.7 ± 0.2 | 0.9 ± 0.2 | 0.7 ± 0.1 | 0.9 ± 0.0 | 0.8 ± 0.1 | 0.9 ± 0.1 | 0.6 ± 0.1 | 0.7 ± 0.1 | 0.6 ± 0.0 | 0.6 ± 0.1 |
| 6 | 4.7 | 261.1 | 0.4 ± 0.0 | 0.5 ± 0.0 | 0.8 ± 0.0 | 0.8 ± 0.0 | 0.6 ± 0.1 | 0.6 ± 0.0 | 0.3 ± 0.0 | 0.3 ± 0.0 | 0.9 ± 0.0 | 0.8 ± 0.1 |
| 7 | 14.4 | 333.1 | 1.2 ± 0.2 | 1.4 ± 0.0 | 1.0 ± 0.0 | 1.1 ± 0.0 | 1.2 ± 0.0 | 1.5 ± 0.1 | 1.0 ± 0.1 | 1.2 ± 0.0 | 0.8 ± 0.1 | 0.9 ± 0.1 |
| 8 | 16.3 | 333.1 | 1.8 ± 0.0 | 2.0 ± 0.1 | 1.2 ± 0.1 | 1.2 ± 0.0 | 1.4 ± 0.1 | 1.4 ± 0.2 | 1.5 ± 0.3 | 1.6 ± 0.1 | 0.9 ± 0.1 | 0.7 ± 0.1 |
| 9 | 18.6 | 361.1 | 1.2 ± 0.1 | 1.3 ± 0.3 | 1.9 ± 0.0 | 1.7 ± 0.1 | 1.3 ± 0.2 | 1.2 ± 0.1 | 1.0 ± 0.0 | 0.9 ± 0.2 | 0.8 ± 0.1 | 0.7 ± 0.0 |
| 10 | 23.6 | 375.1 | 1.4 ± 0.1 | 1.4 ± 0.1 | 1.6 ± 0.1 | 1.6 ± 0.0 | 1.1 ± 0.0 | 1.1 ± 0.0 | 1.1 ± 0.1 | 1.0 ± 0.1 | 0.8 ± 0.1 | 0.8 ± 0.1 |
| 11 | 17.9 | 377.1 | 2.1 ± 0.2 | 2.4 ± 0.0 | 0.8 ± 0.1 | 1.1 ± 0.0 | 1.2 ± 0.0 | 1.3 ± 0.1 | 1.2 ± 0.1 | 1.3 ± 0.1 | 0.8 ± 0.0 | 1.0 ± 0.0 |
| 12 | 23.1 | 382.2 | 0.7 ± 0.1 | 0.8 ± 0.1 | 2.3 ± 0.3 | 2.2 ± 0.3 | 0.6 ± 0.1 | 0.7 ± 0.0 | 0.3 ± 0.0 | 0.3 ± 0.0 | 0.4 ± 0.0 | 0.4 ± 0.0 |
| 13 | 26.6 | 403.1 | 1.3 ± 0.2 | 1.1 ± 0.1 | 0.7 ± 0.1 | 0.8 ± 0.1 | 1.1 ± 0.1 | 0.9 ± 0.2 | 1.3 ± 0.3 | 1.0 ± 0.2 | 0.6 ± 0.0 | 0.6 ± 0.0 |
| 14 | 17.1 | 405.1 | 1.3 ± 0.1 | 1.3 ± 0.1 | 1.2 ± 0.2 | 1.1 ± 0.0 | 1.0 ± 0.0 | 1.2 ± 0.0 | 1.1 ± 0.0 | 1.1 ± 0.0 | 1.0 ± 0.1 | 0.9 ± 0.0 |
| 15 | 15.3 | 407.1 | 1.7 ± 0.0 | 1.9 ± 0.2 | 1.2 ± 0.0 | 1.4 ± 0.0 | 1.4 ± 0.0 | 1.3 ± 0.1 | 1.4 ± 0.2 | 1.3 ± 0.2 | 1.5 ± 0.1 | 1.3 ± 0.0 |
| 16 | 16.4 | 407.1 | 1.1 ± 0.2 | 1.4 ± 0.2 | 0.8 ± 0.1 | 0.7 ± 0.1 | 1.1 ± 0.0 | 1.0 ± 0.1 | 0.8 ± 0.1 | 0.9 ± 0.0 | 1.2 ± 0.1 | 1.0 ± 0.1 |
| 17 | 16.6 | 421.1 | 1.4 ± 0.0 | 1.3 ± 0.2 | 1.0 ± 0.2 | 1.1 ± 0.1 | 1.1 ± 0.1 | 1.1 ± 0.1 | 1.2 ± 0.2 | 1.2 ± 0.0 | 0.9 ± 0.1 | 0.7 ± 0.0 |
| 18 | 15.8 | 298.0 | 0.9 ± 0.0 | 0.9 ± 0.0 | 3.4 ± 0.5 | 3.5 ± 0.6 | 2.1 ± 0.2 | 1.8 ± 0.5 | 0.6 ± 0.2 | 0.6 ± 0.0 | 0.1 ± 0.0 | 0.0 ± 0.0 |
| 19 | 16.8 | 303.1 | 1.0 ± 0.1 | 1.0 ± 0.0 | 0.8 ± 0.1 | 0.8 ± 0.1 | 1.0 ± 0.0 | 0.8 ± 0.0 | 1.1 ± 0.0 | 0.9 ± 0.0 | 0.5 ± 0.1 | 0.5 ± 0.1 |
| 20 | 14.3 | 308.1 | 1.0 ± 0.1 | 0.9 ± 0.0 | 0.7 ± 0.0 | 0.6 ± 0.0 | 0.9 ± 0.1 | 0.7 ± 0.1 | 1.1 ± 0.1 | 0.9 ± 0.0 | 0.5 ± 0.1 | 0.4 ± 0.1 |
| 21 | 11.8 | 348.1 | 0.8 ± 0.2 | 0.8 ± 0.0 | 1.0 ± 0.0 | 0.8 ± 0.0 | 1.1 ± 0.0 | 0.9 ± 0.0 | 1.0 ± 0.1 | 0.8 ± 0.0 | 0.7 ± 0.1 | 0.6 ± 0.1 |
| 22 | 17.8 | 361.1 | 0.9 ± 0.1 | 0.9 ± 0.1 | 1.1 ± 0.2 | 0.9 ± 0.2 | 0.9 ± 0.0 | 0.7 ± 0.1 | 0.9 ± 0.1 | 1.1 ± 0.0 | 0.7 ± 0.1 | 0.6 ± 0.1 |
| 23 | 7.0 | 362.1 | 2.0 ± 0.3 | 1.9 ± 0.3 | 0.9 ± 0.2 | 0.8 ± 0.0 | 1.4 ± 0.3 | 1.1 ± 0.2 | 1.2 ± 0.0 | 1.4 ± 0.1 | 0.8 ± 0.0 | 0.8 ± 0.0 |
| 24 | 14.6 | 363.1 | 2.0 ± 0.4 | 1.7 ± 0.1 | 1.5 ± 0.0 | 1.2 ± 0.1 | 1.5 ± 0.1 | 1.5 ± 0.2 | 1.4 ± 0.3 | 1.1 ± 0.1 | 1.3 ± 0.1 | 1.1 ± 0.2 |
| 25 | 16.1 | 365.1 | 1.1 ± 0.1 | 1.2 ± 0.1 | 1.2 ± 0.1 | 1.1 ± 0.0 | 1.2 ± 0.1 | 1.1 ± 0.1 | 1.0 ± 0.0 | 1.0 ± 0.0 | 0.7 ± 0.0 | 0.7 ± 0.0 |
| 27 | 16.4 | 371.1 | 1.0 ± 0.2 | 0.9 ± 0.1 | 0.7 ± 0.0 | 0.8 ± 0.0 | 1.0 ± 0.1 | 0.9 ± 0.1 | 0.7 ± 0.1 | 0.6 ± 0.1 | 0.7 ± 0.1 | 0.5 ± 0.0 |
| 28 | 17.1 | 371.1 | 1.0 ± 0.2 | 1.0 ± 0.1 | 0.8 ± 0.2 | 0.6 ± 0.0 | 1.0 ± 0.0 | 0.8 ± 0.1 | 1.0 ± 0.1 | 0.9 ± 0.2 | 0.5 ± 0.0 | 0.5 ± 0.1 |
| 29 | 21.8 | 376.1 | 1.7 ± 0.2 | 1.5 ± 0.0 | 1.2 ± 0.2 | 0.9 ± 0.0 | 1.3 ± 0.1 | 1.2 ± 0.1 | 1.5 ± 0.1 | 1.4 ± 0.0 | 1.1 ± 0.1 | 0.9 ± 0.0 |
| 30 | 25.4 | 376.2 | 1.2 ± 0.1 | 1.2 ± 0.2 | 1.2 ± 0.2 | 1.1 ± 0.0 | 1.7 ± 0.1 | 1.9 ± 0.0 | 1.6 ± 0.2 | 1.4 ± 0.2 | 1.0 ± 0.2 | 0.9 ± 0.0 |
| 31 | 16.5 | 377.1 | 2.8 ± 0.1 | 3.1 ± 0.1 | 1.2 ± 0.0 | 1.2 ± 0.1 | 1.4 ± 0.2 | 1.3 ± 0.1 | 1.4 ± 0.0 | 1.3 ± 0.0 | 0.9 ± 0.0 | 0.9 ± 0.0 |
| 32 | 21.4 | 379.1 | 0.3 ± 0.0 | 0.5 ± 0.1 | 0.5 ± 0.0 | 0.6 ± 0.0 | 0.4 ± 0.1 | 0.3 ± 0.0 | 0.3 ± 0.1 | 0.5 ± 0.0 | 0.4 ± 0.0 | 0.5 ± 0.0 |
| 33 | 6.3 | 380.1 | 0.4 ± 0.0 | 0.4 ± 0.1 | 1.0 ± 0.1 | 1.0 ± 0.0 | 0.2 ± 0.0 | 0.2 ± 0.0 | 0.7 ± 0.0 | 0.6 ± 0.0 | 0.7 ± 0.0 | 0.6 ± 0.0 |
| 34 | 17.5 | 389.1 | 1.2 ± 0.2 | 1.4 ± 0.0 | 1.3 ± 0.2 | 1.2 ± 0.2 | 1.5 ± 0.3 | 1.5 ± 0.0 | 1.2 ± 0.1 | 0.9 ± 0.1 | 0.7 ± 0.1 | 0.7 ± 0.1 |
| 35 | 24.5 | 389.1 | 1.1 ± 0.2 | 0.8 ± 0.1 | 1.1 ± 0.1 | 0.9 ± 0.1 | 0.8 ± 0.1 | 0.7 ± 0.2 | 0.8 ± 0.2 | 0.8 ± 0.1 | 0.9 ± 0.1 | 0.8 ± 0.1 |
| 36 | 16.1 | 391.1 | 1.3 ± 0.1 | 1.3 ± 0.0 | 1.0 ± 0.1 | 1.0 ± 0.1 | 1.0 ± 0.1 | 0.9 ± 0.1 | 1.2 ± 0.0 | 1.1 ± 0.0 | 0.9 ± 0.0 | 0.9 ± 0.1 |
| 37 | 17.0 | 391.1 | 1.8 ± 0.1 | 1.6 ± 0.2 | 1.3 ± 0.2 | 1.2 ± 0.2 | 2.8 ± 0.1 | 2.8 ± 0.4 | 1.0 ± 0.2 | 1.0 ± 0.2 | 0.8 ± 0.2 | 0.7 ± 0.1 |
| 38 | 18.4 | 391.1 | 2.1 ± 0.2 | 2.0 ± 0.1 | 1.2 ± 0.1 | 1.3 ± 0.2 | 1.3 ± 0.3 | 1.4 ± 0.0 | 1.1 ± 0.0 | 0.8 ± 0.1 | 0.9 ± 0.0 | 0.7 ± 0.1 |
| 39 | 21.1 | 391.1 | 3.0 ± 0.3 | 2.7 ± 0.6 | 1.2 ± 0.1 | 1.0 ± 0.1 | 1.4 ± 0.2 | 1.3 ± 0.3 | 1.2 ± 0.3 | 1.2 ± 0.1 | 1.1 ± 0.0 | 0.9 ± 0.1 |
| 40 | 27.9 | 401.2 | 3.0 ± 0.4 | 2.7 ± 0.0 | 2.3 ± 0.4 | 2.4 ± 0.5 | 4.3 ± 0.1 | 4.4 ± 0.6 | 1.5 ± 0.0 | 1.3 ± 0.3 | 0.8 ± 0.1 | 0.7 ± 0.1 |
| 41 | 28.5 | 403.2 | 2.9 ± 0.3 | 2.8 ± 0.0 | 2.8 ± 0.2 | 2.8 ± 0.3 | 1.4 ± 0.2 | 1.5 ± 0.0 | 1.9 ± 0.1 | 1.6 ± 0.1 | 1.3 ± 0.1 | 1.3 ± 0.0 |
| 42 | 29.1 | 403.1 | 1.0 ± 0.1 | 1.0 ± 0.0 | 0.9 ± 0.2 | 0.7 ± 0.2 | 1.1 ± 0.1 | 1.0 ± 0.2 | 1.3 ± 0.2 | 1.0 ± 0.2 | 0.9 ± 0.0 | 0.7 ± 0.1 |
| 43 | 16.9 | 407.1 | 2.1 ± 0.4 | 1.7 ± 0.2 | 1.3 ± 0.0 | 1.5 ± 0.1 | 1.5 ± 0.1 | 1.6 ± 0.1 | 1.6 ± 0.3 | 1.5 ± 0.0 | 1.2 ± 0.3 | 1.3 ± 0.1 |
| 44 | 17.2 | 407.1 | 1.2 ± 0.0 | 1.3 ± 0.3 | 1.3 ± 0.1 | 1.1 ± 0.1 | 1.2 ± 0.0 | 1.1 ± 0.3 | 1.2 ± 0.1 | 1.2 ± 0.1 | 1.1 ± 0.1 | 1.2 ± 0.1 |
| 45 | 23.3 | 412.1 | 0.5 ± 0.1 | 0.8 ± 0.2 | 1.8 ± 0.1 | 1.6 ± 0.1 | 0.5 ± 0.1 | 0.6 ± 0.0 | 0.5 ± 0.1 | 0.5 ± 0.1 | 0.6 ± 0.1 | 0.5 ± 0.1 |
| 46 | 17.2 | 415.1 | 1.0 ± 0.1 | 0.9 ± 0.0 | 0.8 ± 0.1 | 0.7 ± 0.0 | 1.0 ± 0.2 | 0.9 ± 0.0 | 0.9 ± 0.0 | 0.9 ± 0.1 | 0.5 ± 0.1 | 0.5 ± 0.0 |
| 47 | 16.7 | 416.1 | 1.6 ± 0.1 | 1.5 ± 0.0 | 1.2 ± 0.0 | 1.0 ± 0.1 | 1.1 ± 0.1 | 1.1 ± 0.0 | 1.1 ± 0.0 | 1.0 ± 0.0 | 0.7 ± 0.1 | 0.7 ± 0.1 |
| 48 | 30.2 | 417.2 | 1.2 ± 0.1 | 1.3 ± 0.0 | 0.8 ± 0.0 | 0.8 ± 0.1 | 1.2 ± 0.1 | 1.2 ± 0.1 | 1.1 ± 0.0 | 1.4 ± 0.0 | 1.0 ± 0.0 | 1.0 ± 0.0 |
| 49 | 15.8 | 419.1 | 0.8 ± 0.0 | 0.8 ± 0.0 | 3.5 ± 0.6 | 3.8 ± 0.1 | 1.8 ± 0.2 | 1.5 ± 0.0 | 0.7 ± 0.1 | 0.7 ± 0.1 | LOQ | LOQ |
| 50 | 18.9 | 419.1 | 1.4 ± 0.1 | 1.4 ± 0.1 | 1.0 ± 0.0 | 0.9 ± 0.0 | 1.2 ± 0.3 | 1.1 ± 0.1 | 1.6 ± 0.2 | 1.3 ± 0.1 | 1.0 ± 0.1 | 0.9 ± 0.1 |
| 51 | 27.6 | 419.2 | 1.1 ± 0.0 | 1.3 ± 0.2 | 1.2 ± 0.2 | 1.3 ± 0.0 | 1.2 ± 0.1 | 1.0 ± 0.2 | 0.9 ± 0.2 | 0.7 ± 0.1 | 2.9 ± 0.2 | 3.1 ± 0.2 |
| 52 | 5.8 | 420.1 | 2.4 ± 0.2 | 2.6 ± 0.1 | 0.8 ± 0.1 | 0.8 ± 0.1 | 1.0 ± 0.1 | 1.0 ± 0.0 | 1.4 ± 0.0 | 1.4 ± 0.1 | 0.9 ± 0.1 | 1.0 ± 0.1 |
| 53 | 11.9 | 423.1 | 2.3 ± 0.5 | 2.2 ± 0.4 | 1.7 ± 0.1 | 1.5 ± 0.2 | 1.6 ± 0.2 | 1.7 ± 0.1 | 1.4 ± 0.2 | 1.5 ± 0.3 | 1.3 ± 0.3 | 1.2 ± 0.3 |
| 54 | 12.7 | 423.1 | 1.4 ± 0.0 | 1.7 ± 0.2 | 1.6 ± 0.4 | 1.8 ± 0.4 | 0.9 ± 0.1 | 0.9 ± 0.1 | 1.1 ± 0.1 | 1.0 ± 0.1 | 1.0 ± 0.0 | 1.1 ± 0.2 |
| 55 | 17.1 | 423.1 | 1.2 ± 0.1 | 1.2 ± 0.3 | 1.2 ± 0.2 | 1.1 ± 0.1 | 0.9 ± 0.0 | 1.1 ± 0.1 | 1.0 ± 0.2 | 1.2 ± 0.1 | 0.9 ± 0.1 | 0.9 ± 0.1 |
| 56 | 26.5 | 423.1 | 1.4 ± 0.2 | 1.5 ± 0.1 | 2.8 ± 0.1 | 2.4 ± 0.0 | 3.4 ± 0.2 | 3.3 ± 0.1 | 1.4 ± 0.1 | 1.3 ± 0.0 | 1.2 ± 0.0 | 1.0 ± 0.1 |
| 57 | 18.1 | 428.1 | 1.4 ± 0.0 | 1.3 ± 0.1 | 0.8 ± 0.2 | 0.8 ± 0.1 | 0.9 ± 0.0 | 1.1 ± 0.1 | 0.8 ± 0.0 | 0.8 ± 0.0 | 0.6 ± 0.0 | 0.7 ± 0.1 |
| 58 | 17.9 | 429.1 | 1.5 ± 0.2 | 1.2 ± 0.2 | 1.0 ± 0.2 | 0.7 ± 0.0 | 1.2 ± 0.1 | 1.0 ± 0.2 | 0.7 ± 0.1 | 0.8 ± 0.2 | 0.6 ± 0.1 | 0.6 ± 0.1 |
| 60 | 33.6 | 429.2 | 0.7 ± 0.0 | 0.7 ± 0.0 | 1.1 ± 0.1 | 1.0 ± 0.0 | 0.9 ± 0.0 | 0.8 ± 0.0 | 0.7 ± 0.0 | 0.6 ± 0.1 | 0.6 ± 0.0 | 0.5 ± 0.1 |
| 61 | 19.8 | 432.1 | 0.8 ± 0.1 | 0.9 ± 0.0 | 1.3 ± 0.3 | 1.2 ± 0.0 | 0.7 ± 0.1 | 0.6 ± 0.0 | 1.2 ± 0.1 | 1.3 ± 0.3 | 0.5 ± 0.1 | 0.6 ± 0.0 |
| 62 | 16.5 | 433.1 | 1.2 ± 0.0 | 1.0 ± 0.1 | 0.8 ± 0.1 | 0.8 ± 0.1 | 1.0 ± 0.0 | 1.0 ± 0.0 | 1.1 ± 0.1 | 1.1 ± 0.1 | 0.6 ± 0.1 | 0.6 ± 0.1 |
| 63 | 17.2 | 433.1 | 1.0 ± 0.0 | 1.1 ± 0.1 | 0.7 ± 0.0 | 0.7 ± 0.0 | 1.0 ± 0.0 | 0.8 ± 0.0 | 1.0 ± 0.1 | 0.9 ± 0.1 | 0.6 ± 0.0 | 0.5 ± 0.0 |
| 64 | 19.1 | 433.1 | 1.4 ± 0.0 | 1.2 ± 0.1 | 0.8 ± 0.0 | 0.8 ± 0.1 | 1.0 ± 0.1 | 1.0 ± 0.2 | 1.4 ± 0.1 | 1.3 ± 0.1 | 0.7 ± 0.0 | 0.7 ± 0.1 |
| 65 | 20.7 | 433.1 | 2.2 ± 0.0 | 2.2 ± 0.4 | 1.5 ± 0.1 | 1.4 ± 0.2 | 1.4 ± 0.2 | 1.5 ± 0.4 | 1.9 ± 0.1 | 1.8 ± 0.3 | 1.0 ± 0.2 | 1.3 ± 0.2 |
| 66 | 22.3 | 433.1 | 1.0 ± 0.2 | 1.3 ± 0.3 | 0.8 ± 0.1 | 0.8 ± 0.0 | 1.0 ± 0.1 | 1.1 ± 0.0 | 1.5 ± 0.1 | 1.6 ± 0.1 | 0.7 ± 0.2 | 0.8 ± 0.1 |
| 67 | 16.4 | 434.1 | 3.9 ± 0.5 | 4.0 ± 0.4 | 0.9 ± 0.1 | 1.0 ± 0.0 | 1.0 ± 0.1 | 1.1 ± 0.0 | 1.3 ± 0.3 | 1.3 ± 0.0 | 0.8 ± 0.1 | 0.8 ± 0.1 |
| 68 | 16.5 | 435.1 | 1.3 ± 0.1 | 1.3 ± 0.3 | 1.1 ± 0.1 | 1.0 ± 0.1 | 0.9 ± 0.2 | 0.9 ± 0.0 | 1.3 ± 0.1 | 1.1 ± 0.0 | 0.7 ± 0.0 | 0.8 ± 0.1 |
| 69 | 17.3 | 435.1 | 1.5 ± 0.2 | 1.5 ± 0.0 | 1.0 ± 0.1 | 0.9 ± 0.2 | 1.7 ± 0.3 | 1.7 ± 0.0 | 1.4 ± 0.3 | 1.6 ± 0.2 | 1.0 ± 0.2 | 0.8 ± 0.1 |
| 70 | 19.2 | 437.1 | 1.1 ± 0.1 | 1.2 ± 0.0 | 1.4 ± 0.1 | 1.4 ± 0.3 | 0.6 ± 0.1 | 0.6 ± 0.0 | 0.8 ± 0.1 | 0.7 ± 0.2 | 0.7 ± 0.0 | 0.9 ± 0.0 |
| 71 | 21.4 | 444.1 | 1.5 ± 0.1 | 1.3 ± 0.1 | 0.6 ± 0.1 | 0.5 ± 0.1 | 1.0 ± 0.2 | 0.9 ± 0.0 | 1.0 ± 0.1 | 1.1 ± 0.1 | 0.6 ± 0.0 | 0.4 ± 0.0 |
| 72 | 33.8 | 447.2 | 0.9 ± 0.1 | 0.7 ± 0.0 | 0.9 ± 0.0 | 1.1 ± 0.1 | 0.7 ± 0.1 | 0.8 ± 0.0 | 0.7 ± 0.1 | 0.7 ± 0.0 | 1.1 ± 0.0 | 0.9 ± 0.2 |
| 73 | 16.2 | 448.2 | 1.7 ± 0.2 | 1.8 ± 0.1 | 1.1 ± 0.0 | 1.1 ± 0.1 | 1.0 ± 0.0 | 1.1 ± 0.2 | 1.0 ± 0.1 | 1.0 ± 0.1 | 0.7 ± 0.0 | 0.8 ± 0.1 |
| 74 | 19.2 | 449.2 | 1.2 ± 0.0 | 1.5 ± 0.1 | 1.0 ± 0.1 | 1.1 ± 0.2 | 1.0 ± 0.0 | 1.1 ± 0.1 | 0.8 ± 0.2 | 1.1 ± 0.1 | 0.6 ± 0.0 | 0.7 ± 0.0 |
| 75 | 14.2 | 450.1 | 2.4 ± 0.3 | 2.3 ± 0.2 | 0.8 ± 0.1 | 0.9 ± 0.0 | 0.9 ± 0.0 | 1.1 ± 0.1 | 1.2 ± 0.1 | 1.2 ± 0.1 | 0.8 ± 0.0 | 0.8 ± 0.1 |
| 76 | 36.6 | 453.2 | 0.6 ± 0.1 | 0.5 ± 0.0 | 0.7 ± 0.1 | 1.0 ± 0.0 | 0.8 ± 0.0 | 0.7 ± 0.1 | 1.0 ± 0.2 | 1.0 ± 0.2 | 0.4 ± 0.0 | 0.3 ± 0.0 |
| 77 | 27.9 | 455.1 | 0.4 ± 0.0 | 0.4 ± 0.1 | 0.9 ± 0.0 | 0.7 ± 0.1 | 2.0 ± 0.3 | 1.9 ± 0.1 | 0.5 ± 0.0 | 0.4 ± 0.1 | 0.3 ± 0.0 | 0.2 ± 0.0 |
| 78 | 5.9 | 459.1 | 1.2 ± 0.0 | 1.2 ± 0.3 | 0.6 ± 0.1 | 0.8 ± 0.1 | 0.8 ± 0.1 | 0.9 ± 0.0 | 1.0 ± 0.0 | 1.0 ± 0.1 | 0.7 ± 0.1 | 0.7 ± 0.0 |
| 79 | 24.5 | 459.2 | 1.4 ± 0.2 | 1.5 ± 0.1 | 1.2 ± 0.0 | 1.2 ± 0.0 | 1.2 ± 0.0 | 1.3 ± 0.0 | 1.0 ± 0.1 | 0.9 ± 0.0 | 0.7 ± 0.1 | 0.8 ± 0.0 |
| 80 | 26.0 | 461.2 | 3.8 ± 0.4 | 3.5 ± 0.2 | 1.7 ± 0.1 | 1.5 ± 0.0 | 1.8 ± 0.0 | 1.6 ± 0.2 | 1.7 ± 0.3 | 1.6 ± 0.2 | 1.0 ± 0.0 | 1.0 ± 0.0 |
| 81 | 16.7 | 462.1 | 1.4 ± 0.2 | 1.5 ± 0.3 | 1.3 ± 0.3 | 1.2 ± 0.0 | 0.9 ± 0.0 | 1.2 ± 0.2 | 1.0 ± 0.2 | 1.3 ± 0.1 | 1.1 ± 0.1 | 0.8 ± 0.0 |
| 82 | 20.9 | 462.2 | 1.3 ± 0.1 | 1.4 ± 0.0 | 0.3 ± 0.1 | 0.5 ± 0.1 | 1.0 ± 0.1 | 1.2 ± 0.0 | 0.8 ± 0.1 | 0.7 ± 0.0 | 0.4 ± 0.1 | 0.5 ± 0.1 |
| 83 | 19.8 | 472.1 | 1.3 ± 0.0 | 1.1 ± 0.1 | 0.8 ± 0.0 | 0.8 ± 0.1 | 1.0 ± 0.1 | 1.0 ± 0.1 | 0.9 ± 0.1 | 0.9 ± 0.0 | 0.6 ± 0.0 | 0.6 ± 0.0 |
| 84 | 7.1 | 477.2 | 1.8 ± 0.2 | 1.9 ± 0.1 | 0.8 ± 0.0 | 0.8 ± 0.1 | 1.2 ± 0.1 | 1.2 ± 0.0 | 1.2 ± 0.0 | 1.2 ± 0.1 | 0.9 ± 0.1 | 0.8 ± 0.1 |
| 85 | 15.0 | 478.1 | 0.7 ± 0.0 | 0.7 ± 0.0 | 0.7 ± 0.0 | 0.8 ± 0.1 | 1.0 ± 0.1 | 1.0 ± 0.0 | 1.0 ± 0.1 | 0.9 ± 0.0 | 0.6 ± 0.0 | 0.6 ± 0.0 |
| 86 | 31.1 | 482.2 | 1.1 ± 0.1 | 1.3 ± 0.2 | 1.4 ± 0.0 | 1.4 ± 0.1 | 1.3 ± 0.2 | 1.1 ± 0.3 | 1.6 ± 0.2 | 1.3 ± 0.1 | 0.9 ± 0.0 | 1.1 ± 0.0 |
| 87 | 16.1 | 490.1 | 1.0 ± 0.2 | 1.1 ± 0.0 | 1.1 ± 0.2 | 0.9 ± 0.0 | 1.0 ± 0.1 | 1.0 ± 0.1 | 0.9 ± 0.2 | 0.8 ± 0.1 | 0.8 ± 0.0 | 0.6 ± 0.0 |
| 88 | 17.2 | 490.1 | 1.2 ± 0.0 | 1.1 ± 0.0 | 0.8 ± 0.0 | 0.7 ± 0.1 | 0.9 ± 0.0 | 1.0 ± 0.0 | 0.7 ± 0.1 | 0.8 ± 0.0 | 0.8 ± 0.2 | 0.7 ± 0.1 |
| 89 | 18.2 | 490.1 | 1.3 ± 0.1 | 1.3 ± 0.3 | 0.8 ± 0.0 | 0.9 ± 0.1 | 1.1 ± 0.1 | 1.0 ± 0.0 | 0.9 ± 0.0 | 0.8 ± 0.0 | 0.6 ± 0.2 | 0.6 ± 0.0 |
| 90 | 11.2 | 491.2 | 1.1 ± 0.1 | 1.2 ± 0.0 | 1.0 ± 0.1 | 1.1 ± 0.0 | 0.9 ± 0.1 | 1.0 ± 0.1 | 1.4 ± 0.1 | 1.4 ± 0.2 | 0.9 ± 0.1 | 0.8 ± 0.0 |
| 91 | 18.6 | 506.2 | 1.9 ± 0.3 | 1.6 ± 0.3 | 1.2 ± 0.3 | 0.9 ± 0.2 | 1.1 ± 0.0 | 0.9 ± 0.0 | 1.0 ± 0.2 | 1.2 ± 0.0 | 0.8 ± 0.1 | 0.9 ± 0.1 |
| 92 | 21.2 | 506.2 | 1.2 ± 0.1 | 1.1 ± 0.3 | 0.9 ± 0.2 | 0.7 ± 0.0 | 1.3 ± 0.0 | 1.4 ± 0.1 | 0.9 ± 0.1 | 0.9 ± 0.2 | 0.6 ± 0.0 | 0.6 ± 0.0 |
| 93 | 27.1 | 528.2 | 1.1 ± 0.1 | 1.0 ± 0.2 | 1.3 ± 0.1 | 1.2 ± 0.1 | 0.9 ± 0.1 | 1.2 ± 0.2 | 0.9 ± 0.1 | 1.2 ± 0.1 | 1.0 ± 0.1 | 0.8 ± 0.2 |
| 94 | 25.5 | 529.2 | 1.6 ± 0.1 | 1.4 ± 0.2 | 1.4 ± 0.1 | 1.2 ± 0.3 | 1.2 ± 0.3 | 1.1 ± 0.3 | 1.3 ± 0.0 | 1.4 ± 0.1 | 1.4 ± 0.1 | 1.2 ± 0.1 |
| 95 | 17.3 | 533.2 | 0.9 ± 0.0 | 1.0 ± 0.1 | 0.7 ± 0.0 | 0.7 ± 0.0 | 0.8 ± 0.0 | 0.9 ± 0.0 | 1.0 ± 0.0 | 1.0 ± 0.0 | 0.7 ± 0.0 | 0.8 ± 0.1 |
| 97 | 14.7 | 546.2 | 0.9 ± 0.2 | 1.0 ± 0.1 | 0.8 ± 0.0 | 0.8 ± 0.1 | 0.9 ± 0.1 | 0.9 ± 0.1 | 0.7 ± 0.0 | 0.9 ± 0.0 | 0.8 ± 0.0 | 0.7 ± 0.0 |
| 98 | 14.0 | 549.2 | 1.0 ± 0.1 | 1.0 ± 0.2 | 0.9 ± 0.0 | 1.1 ± 0.0 | 1.0 ± 0.0 | 1.0 ± 0.1 | 1.3 ± 0.2 | 1.4 ± 0.4 | 0.7 ± 0.2 | 0.7 ± 0.1 |
| 99 | 16.9 | 551.2 | 1.1 ± 0.1 | 1.2 ± 0.3 | 0.9 ± 0.2 | 1.0 ± 0.0 | 1.0 ± 0.2 | 0.9 ± 0.1 | 1.0 ± 0.2 | 1.0 ± 0.1 | 0.8 ± 0.1 | 0.8 ± 0.2 |
| 100 | 18.0 | 563.2 | 4.7 ± 0.2 | 4.5 ± 0.1 | 0.7 ± 0.1 | 0.7 ± 0.0 | 0.9 ± 0.0 | 0.8 ± 0.0 | 1.7 ± 0.1 | 1.4 ± 0.1 | 0.5 ± 0.0 | 0.6 ± 0.1 |
| 101 | 18.4 | 577.2 | 0.9 ± 0.2 | 0.9 ± 0.1 | 0.8 ± 0.2 | 1.1 ± 0.3 | 1.1 ± 0.2 | 1.0 ± 0.2 | 1.0 ± 0.2 | 1.2 ± 0.1 | 0.9 ± 0.2 | 0.7 ± 0.2 |
| 102 | 20.1 | 577.2 | 5.0 ± 0.5 | 4.8 ± 0.5 | 0.7 ± 0.1 | 0.7 ± 0.0 | 1.0 ± 0.3 | 1.0 ± 0.0 | 1.0 ± 0.2 | 1.1 ± 0.1 | 0.4 ± 0.0 | 0.5 ± 0.1 |

Table S3. List of log_2_ (fold change) and –log_10_ (P value) of thiols from 5 cancer urines.

| NO. | Time  (min) | *m/z* | **Nasopharyngeal**  **cancer** | | **Esophagus**  **cancer** | | **Gastric**  **cancer** | | **Lymph**  **cancer** | | **Lung**  **cancer** | |
| --- | --- | --- | --- | --- | --- | --- | --- | --- | --- | --- | --- | --- |
|  |  |  | log_2_ (fold  change) | –log_10_ (P  value) | log_2_ (fold  change) | –log_10_ (P  value) | log_2_ (fold  change) | –log_10_ (P  value) | log_2_ (fold  change) | –log_10_ (P  value) | log_2_ (fold  change) | –log_10_ (P  value) |
| 1 | 3.7 | 305.1 | 0.6 | 1.9 | -0.4 | 1.3 | 0.4 | 1.7 | 0.4 | 1.3 | -0.4 | 1.9 |
| 2 | 4.4 | 319.1 | 1.2 | 6.8 | 0.6 | 3.1 | 0.7 | 6.0 | 0.5 | 4.6 | -0.4 | 2.8 |
| 3 | 16.1 | 347.1 | 0.1 | 1.4 | 0.1 | 2.1 | 0.1 | 2.2 | 0.0 | 0.0 | -0.4 | 5.2 |
| 4 | 7.9 | 434.1 | -0.4 | 1.7 | -0.2 | 1.1 | 1.3 | 4.1 | -0.1 | 0.2 | -0.6 | 3.8 |
| 5 | 8.6 | 491.2 | -0.3 | 1.5 | -0.3 | 1.6 | -0.3 | 1.7 | -0.6 | 0.9 | -0.7 | 4.4 |
| 6 | 4.7 | 261.1 | -1.1 | 2.4 | -0.3 | 2.3 | -0.8 | 4.5 | -1.7 | 9.1 | -0.3 | 2.1 |
| 7 | 14.4 | 333.1 | 0.4 | 2.1 | 0.1 | 0.4 | 0.4 | 2.9 | 0.2 | 0.9 | -0.2 | 1.3 |
| 8 | 16.3 | 333.1 | 0.9 | 4.5 | 0.2 | 1.3 | 0.5 | 2.6 | 0.7 | 1.8 | -0.3 | 1.3 |
| 9 | 18.6 | 361.1 | 0.3 | 1.0 | 0.9 | 2.4 | 0.3 | 1.8 | -0.1 | 0.2 | -0.4 | 1.7 |
| 10 | 23.6 | 375.1 | 0.5 | 3.2 | 0.7 | 1.9 | 0.2 | 0.9 | 0.1 | 0.2 | -0.3 | 1.8 |
| 11 | 17.9 | 377.1 | 1.2 | 3.9 | -0.1 | 0.7 | 0.3 | 2.0 | 0.3 | 1.9 | -0.2 | 2.0 |
| 12 | 23.1 | 382.2 | -0.5 | 2.0 | 1.2 | 4.8 | -0.6 | 2.4 | -1.7 | 4.2 | -1.4 | 5.5 |
| 13 | 26.6 | 403.1 | 0.3 | 0.8 | -0.4 | 2.4 | 0.0 | 0.0 | 0.2 | 0.6 | -0.7 | 3.6 |
| 14 | 17.1 | 405.1 | 0.4 | 2.5 | 0.2 | 1.3 | 0.1 | 1.2 | 0.1 | 0.8 | -0.1 | 0.5 |
| 15 | 15.3 | 407.1 | 0.8 | 2.3 | 0.4 | 5.0 | 0.4 | 1.5 | 0.4 | 1.2 | 0.5 | 1.5 |
| 16 | 16.4 | 407.1 | 0.4 | 1.9 | -0.4 | 2.8 | 0.1 | 0.7 | -0.2 | 1.3 | 0.2 | 0.7 |
| 17 | 16.6 | 421.1 | 0.4 | 2.2 | 0.0 | 0.2 | 0.1 | 0.6 | 0.2 | 1.3 | -0.3 | 1.1 |
| 18 | 15.8 | 298.0 | -0.2 | 1.1 | 1.8 | 6.1 | 1.0 | 3.9 | -0.7 | 2.0 | -4.6 | 5.8 |
| 19 | 16.8 | 303.1 | 0.0 | 0.0 | -0.4 | 1.4 | -0.1 | 0.1 | 0.0 | 0.0 | -0.9 | 2.4 |
| 20 | 14.3 | 308.1 | -0.1 | 0.3 | -0.7 | 3.9 | -0.3 | 1.7 | 0.0 | 0.0 | -1.1 | 4.5 |
| 21 | 11.8 | 348.1 | -0.3 | 1.4 | -0.1 | 0.4 | 0.0 | 0.1 | -0.1 | 0.3 | -0.6 | 2.4 |
| 22 | 17.8 | 361.1 | -0.2 | 0.6 | 0.0 | 0.1 | -0.3 | 1.6 | 0.0 | 0.1 | -0.6 | 3.7 |
| 23 | 7.0 | 362.1 | 1.0 | 2.1 | -0.2 | 0.9 | 0.3 | 1.2 | 0.4 | 1.2 | -0.3 | 1.9 |
| 24 | 14.6 | 363.1 | 0.9 | 2.4 | 0.4 | 2.3 | 0.6 | 3.2 | 0.3 | 1.6 | 0.2 | 1.4 |
| 25 | 16.1 | 365.1 | 0.2 | 1.5 | 0.2 | 1.9 | 0.2 | 2.2 | 0.0 | 0.0 | -0.5 | 4.7 |
| 27 | 16.4 | 371.1 | -0.1 | 0.3 | -0.4 | 1.9 | -0.1 | 0.2 | -0.6 | 1.4 | -0.8 | 1.6 |
| 28 | 17.1 | 371.1 | 0.0 | 0.0 | -0.5 | 2.1 | -0.2 | 0.6 | -0.1 | 0.4 | -1.0 | 3.9 |
| 29 | 21.8 | 376.1 | 0.7 | 1.9 | 0.1 | 0.1 | 0.4 | 1.2 | 0.5 | 3.2 | 0.0 | 0.0 |
| 30 | 25.4 | 376.2 | 0.3 | 0.9 | 0.2 | 0.5 | 0.9 | 3.3 | 0.6 | 2.7 | -0.1 | 0.2 |
| 31 | 16.5 | 377.1 | 1.6 | 3.0 | 0.2 | 1.4 | 0.5 | 2.1 | 0.5 | 1.8 | -0.2 | 1.5 |
| 32 | 21.4 | 379.1 | -1.3 | 3.7 | -1.0 | 4.6 | -1.4 | 2.0 | -1.4 | 4.9 | -1.1 | 3.3 |
| 33 | 6.3 | 380.1 | -1.5 | 6.7 | 0.0 | 0.3 | -2.4 | 9.0 | -0.7 | 2.9 | -0.5 | 4.1 |
| 34 | 17.5 | 389.1 | 0.4 | 1.5 | 0.3 | 1.0 | 0.5 | 2.7 | 0.1 | 0.3 | -0.6 | 2.6 |
| 35 | 24.5 | 389.1 | 0.0 | 0.2 | 0.0 | 0.1 | -0.5 | 1.7 | -0.3 | 2.0 | -0.3 | 1.0 |
| 36 | 16.1 | 391.1 | 0.4 | 2.0 | 0.0 | 0.0 | 0.0 | 0.1 | 0.2 | 0.7 | -0.1 | 0.5 |
| 37 | 17.0 | 391.1 | 0.8 | 3.3 | 0.3 | 1.3 | 1.5 | 5.8 | 0.0 | 0.2 | -0.4 | 1.4 |
| 38 | 18.4 | 391.1 | 1.1 | 4.1 | 0.3 | 1.5 | 0.4 | 1.9 | -0.1 | 0.3 | -0.3 | 0.9 |
| 39 | 21.1 | 391.1 | 1.5 | 5.3 | 0.1 | 0.5 | 0.4 | 1.6 | 0.3 | 1.2 | 0.0 | 0.1 |
| 40 | 27.9 | 401.2 | 1.5 | 3.5 | 1.2 | 4.8 | 2.1 | 5.3 | 0.5 | 2.6 | -0.4 | 1.4 |
| 41 | 28.5 | 403.2 | 1.5 | 3.1 | 1.5 | 3.3 | 0.5 | 2.6 | 0.8 | 3.3 | 0.4 | 2.5 |
| 42 | 29.1 | 403.1 | 0.0 | 0.1 | -0.3 | 0.8 | 0.1 | 0.3 | 0.2 | 0.7 | -0.3 | 0.9 |
| 43 | 16.9 | 407.1 | 0.9 | 3.2 | 0.5 | 2.7 | 0.6 | 2.3 | 0.6 | 2.6 | 0.4 | 1.3 |
| 44 | 17.2 | 407.1 | 0.3 | 2.0 | 0.2 | 1.1 | 0.2 | 0.8 | 0.2 | 1.2 | 0.2 | 1.1 |
| 45 | 23.3 | 412.1 | -0.6 | 2.2 | 0.8 | 1.8 | -0.8 | 5.3 | -1.0 | 3.3 | -0.9 | 3.1 |
| 46 | 17.2 | 415.1 | -0.1 | 0.2 | -0.4 | 2.1 | -0.1 | 0.5 | -0.1 | 1.2 | -1.1 | 3.4 |
| 47 | 16.7 | 416.1 | 0.6 | 3.1 | 0.1 | 0.6 | 0.1 | 0.8 | 0.0 | 0.1 | -0.5 | 2.7 |
| 48 | 30.2 | 417.2 | 0.3 | 1.6 | -0.3 | 2.4 | 0.3 | 2.3 | 0.3 | 3.0 | 0.0 | 0.1 |
| 49 | 15.8 | 419.1 | -0.4 | 1.2 | 1.8 | 2.8 | 0.7 | 2.8 | -0.5 | 1.5 | -- | -- |
| 50 | 18.9 | 419.1 | 0.5 | 2.4 | -0.1 | 0.3 | 0.2 | 1.0 | 0.5 | 1.5 | -0.1 | 0.2 |
| 51 | 27.6 | 419.2 | 0.2 | 0.8 | 0.3 | 1.4 | 0.1 | 0.4 | -0.4 | 1.2 | 1.6 | 5.1 |
| 52 | 5.8 | 420.1 | 1.3 | 6.8 | -0.3 | 3.1 | 0.0 | 0.2 | 0.5 | 1.8 | -0.1 | 1.2 |
| 53 | 11.9 | 423.1 | 1.2 | 4.3 | 0.7 | 2.1 | 0.7 | 2.9 | 0.6 | 1.3 | 0.3 | 0.8 |
| 54 | 12.7 | 423.1 | 0.7 | 3.7 | 0.8 | 2.5 | -0.1 | 0.4 | 0.1 | 0.4 | 0.1 | 0.2 |
| 55 | 17.1 | 423.1 | 0.2 | 0.8 | 0.2 | 1.8 | 0.0 | 0.1 | 0.1 | 0.4 | -0.1 | 1.0 |
| 56 | 26.5 | 423.1 | 0.5 | 3.5 | 1.4 | 2.8 | 1.8 | 3.7 | 0.4 | 2.5 | 0.1 | 0.7 |
| 57 | 18.1 | 428.1 | 0.4 | 2.3 | -0.3 | 1.9 | 0.0 | 0.1 | -0.3 | 4.1 | -0.7 | 4.4 |
| 58 | 17.9 | 429.1 | 0.4 | 0.7 | -0.3 | 0.5 | 0.1 | 0.3 | -0.4 | 0.9 | -0.8 | 2.5 |
| 60 | 33.6 | 429.2 | -0.5 | 3.1 | 0.1 | 0.9 | -0.3 | 2.7 | -0.6 | 3.5 | -0.8 | 4.1 |
| 61 | 19.8 | 432.1 | -0.2 | 0.8 | 0.3 | 1.2 | -0.5 | 2.4 | 0.3 | 0.4 | -0.8 | 3.9 |
| 62 | 16.5 | 433.1 | 0.2 | 0.5 | -0.3 | 1.5 | 0.0 | 0.1 | 0.1 | 0.4 | -0.8 | 3.2 |
| 63 | 17.2 | 433.1 | 0.1 | 0.2 | -0.5 | 3.5 | -0.2 | 0.8 | -0.1 | 0.2 | -0.8 | 2.2 |
| 64 | 19.1 | 433.1 | 0.4 | 2.5 | -0.3 | 3.0 | 0.0 | 0.1 | 0.4 | 2.2 | -0.5 | 2.8 |
| 65 | 20.7 | 433.1 | 1.1 | 3.6 | 0.5 | 1.9 | 0.5 | 2.1 | 0.9 | 3.4 | 0.2 | 0.5 |
| 66 | 22.3 | 433.1 | 0.2 | 0.3 | -0.3 | 1.1 | 0.1 | 0.4 | 0.6 | 2.3 | -0.4 | 1.2 |
| 67 | 16.4 | 434.1 | 2.0 | 8.0 | -0.1 | 0.5 | 0.1 | 0.3 | 0.4 | 2.1 | -0.3 | 2.9 |
| 68 | 16.5 | 435.1 | 0.4 | 1.4 | 0.0 | 0.1 | -0.2 | 1.1 | 0.2 | 2.0 | -0.5 | 3.4 |
| 69 | 17.3 | 435.1 | 0.6 | 3.5 | -0.1 | 0.3 | 0.7 | 2.7 | 0.6 | 2.7 | -0.1 | 0.5 |
| 70 | 19.2 | 437.1 | 0.2 | 1.1 | 0.5 | 2.1 | -0.7 | 4.5 | -0.4 | 2.3 | -0.2 | 2.5 |
| 71 | 21.4 | 444.1 | 0.4 | 1.3 | -0.8 | 1.5 | -0.1 | 0.2 | 0.0 | 0.1 | -0.9 | 2.4 |
| 72 | 33.8 | 447.2 | -0.4 | 1.4 | 0.0 | 0.1 | -0.4 | 3.3 | -0.5 | 4.4 | 0.0 | 0.1 |
| 73 | 16.2 | 448.2 | 0.8 | 4.5 | 0.1 | 0.7 | 0.1 | 0.2 | 0.0 | 0.2 | -0.4 | 3.2 |
| 74 | 19.2 | 449.2 | 0.4 | 1.4 | 0.0 | 0.1 | 0.1 | 0.4 | 0.0 | 0.2 | -0.6 | 1.9 |
| 75 | 14.2 | 450.1 | 1.2 | 4.7 | -0.3 | 1.4 | 0.0 | 0.1 | 0.2 | 1.0 | -0.3 | 2.1 |
| 76 | 36.6 | 453.2 | -0.9 | 3.4 | -0.2 | 2.0 | -0.5 | 1.8 | 0.0 | 0.0 | -1.4 | 4.5 |
| 77 | 27.9 | 455.1 | -1.3 | 4.6 | -0.3 | 0.9 | 1.0 | 4.0 | -1.3 | 2.8 | -2.0 | 5.9 |
| 78 | 5.9 | 459.1 | 0.3 | 1.6 | -0.4 | 2.0 | -0.3 | 3.1 | 0.0 | 0.5 | -0.4 | 3.1 |
| 79 | 24.5 | 459.2 | 0.5 | 3.2 | 0.3 | 2.1 | 0.3 | 3.3 | -0.1 | 0.6 | -0.4 | 2.2 |
| 80 | 26.0 | 461.2 | 1.9 | 6.7 | 0.7 | 4.4 | 0.7 | 4.5 | 0.7 | 4.1 | 0.0 | 0.2 |
| 81 | 16.7 | 462.1 | 0.6 | 2.5 | 0.3 | 1.3 | 0.1 | 0.1 | 0.3 | 1.0 | -0.1 | 0.2 |
| 82 | 20.9 | 462.2 | 0.4 | 3.6 | -1.2 | 2.4 | 0.1 | 0.6 | -0.4 | 1.9 | -1.1 | 3.0 |
| 83 | 19.8 | 472.1 | 0.3 | 1.5 | -0.3 | 2.3 | 0.0 | 0.2 | -0.2 | 1.0 | -0.8 | 5.7 |
| 84 | 7.1 | 477.2 | 0.9 | 5.5 | -0.3 | 3.4 | 0.3 | 2.9 | 0.3 | 3.3 | -0.2 | 2.9 |
| 85 | 15.0 | 478.1 | -0.5 | 4.0 | -0.4 | -0.7 | 0.0 | 0.3 | -0.1 | 0.9 | -0.7 | 4.1 |
| 86 | 31.1 | 482.2 | 0.3 | 1.5 | 0.4 | 2.2 | 0.3 | 1.0 | 0.5 | 2.1 | 0.0 | 0.0 |
| 87 | 16.1 | 490.1 | 0.1 | 0.2 | 0.0 | 0.2 | 0.0 | 0.0 | -0.3 | 1.5 | -0.5 | 2.5 |
| 88 | 17.2 | 490.1 | 0.2 | 1.1 | -0.5 | 3.3 | -0.1 | 0.5 | -0.4 | 3.1 | -0.4 | 2.9 |
| 89 | 18.2 | 490.1 | 0.4 | 2.2 | -0.2 | 1.4 | 0.1 | 0.4 | -0.2 | 0.9 | -0.7 | 3.6 |
| 90 | 11.2 | 491.2 | 0.2 | 2.3 | 0.1 | 0.3 | -0.1 | 0.5 | 0.5 | 3.0 | -0.2 | 3.9 |
| 91 | 18.6 | 506.2 | 0.8 | 2.8 | 0.0 | 0.0 | -0.1 | 0.1 | 0.1 | 0.2 | -0.2 | 0.7 |
| 92 | 21.2 | 506.2 | 0.2 | 0.9 | -0.3 | 1.3 | 0.4 | 2.5 | -0.1 | 0.4 | -0.7 | 3.2 |
| 93 | 27.1 | 528.2 | 0.0 | 0.0 | 0.3 | 2.0 | 0.0 | 0.0 | 0.1 | 0.2 | -0.2 | 0.6 |
| 94 | 25.5 | 529.2 | 0.6 | 2.5 | 0.4 | 1.0 | 0.2 | 0.7 | 0.5 | 2.9 | 0.3 | 1.1 |
| 95 | 17.3 | 533.2 | -0.1 | 0.6 | -0.5 | 4.1 | -0.3 | 2.6 | 0.0 | 0.0 | -0.4 | 2.3 |
| 97 | 14.7 | 546.2 | 0.0 | 0.1 | -0.3 | 2.0 | -0.2 | 0.9 | -0.3 | 2.1 | -0.4 | 4.8 |
| 98 | 14.0 | 549.2 | 0.0 | 0.2 | 0.0 | 0.0 | 0.0 | 0.2 | 0.4 | 1.4 | -0.6 | 2.7 |
| 99 | 16.9 | 551.2 | 0.2 | 0.4 | -0.1 | 0.1 | -0.1 | 0.3 | 0.0 | 0.1 | -0.3 | 1.1 |
| 100 | 18.0 | 563.2 | 2.2 | 8.0 | -0.5 | 2.3 | -0.2 | 2.7 | 0.6 | 4.3 | -0.8 | 3.8 |
| 101 | 18.4 | 577.2 | -0.1 | 0.3 | -0.1 | 0.2 | 0.1 | 0.2 | 0.2 | 0.4 | -0.3 | 1.5 |
| 102 | 20.1 | 577.2 | 2.3 | 4.4 | -0.5 | 1.8 | 0.1 | 0.1 | 0.0 | 0.1 | -1.1 | 2.7 |

Table S4. List of the measured peak area ratios (cancer/healthy control) of 99 thiols from 50 cancers and 30 healthy urines by IL-LC-MRM-MS method.

| NO. | Time (min) | *m/z* | Peak area ratio (cancer/healthy control) | |
| --- | --- | --- | --- | --- |
|  |  |  | Forward | Reverse |
| 1 | 3.7 | 305.1 | 0.9 ± 0.0 | 1.0 ± 0.1 |
| 2 | 4.4 | 319.1 | 0.8 ± 0.0 | 0.7 ± 0.0 |
| 3 | 16.1 | 347.1 | 0.8 ± 0.1 | 0.8 ± 0.0 |
| 4 | 7.9 | 434.1 | 1.1 ± 0.1 | 1.1 ± 0.2 |
| 5 | 8.6 | 491.2 | 0.5 ± 0.0 | 0.6 ± 0.0 |
| 6 | 4.7 | 261.1 | 0.6 ± 0.0 | 0.7 ± 0.0 |
| 7 | 14.4 | 333.1 | 0.7 ± 0.1 | 0.8 ± 0.0 |
| 8 | 16.3 | 333.1 | 1.2 ± 0.0 | 1.1 ± 0.0 |
| 9 | 18.6 | 361.1 | 0.9 ± 0.1 | 0.9 ± 0.1 |
| 10 | 23.6 | 375.1 | 0.9 ± 0.1 | 0.8 ± 0.0 |
| 11 | 17.9 | 377.1 | 0.8 ± 0.1 | 0.8 ± 0.0 |
| 12 | 23.1 | 382.2 | 1.3 ± 0.3 | 1.1 ± 0.0 |
| 13 | 26.6 | 403.1 | 0.6 ± 0.1 | 0.7 ± 0.1 |
| 14 | 17.1 | 405.1 | 0.9 ± 0.0 | 0.8 ± 0.0 |
| 15 | 15.3 | 407.1 | 0.9 ± 0.2 | 1.1 ± 0.2 |
| 16 | 16.4 | 407.1 | 0.6 ± 0.0 | 0.5 ± 0.1 |
| 17 | 16.6 | 421.1 | 0.8 ± 0.2 | 0.8 ± 0.2 |
| 18 | 15.8 | 298.0 | 1.3 ± 0.2 | 1.2 ± 0.1 |
| 19 | 16.8 | 303.1 | 0.5 ± 0.0 | 0.6 ± 0.1 |
| 20 | 14.3 | 308.1 | 0.5 ± 0.0 | 0.4 ± 0.0 |
| 21 | 11.8 | 348.1 | 0.8 ± 0.2 | 0.9 ± 0.0 |
| 22 | 17.8 | 361.1 | 0.7 ± 0.1 | 0.6 ± 0.1 |
| 23 | 7.0 | 362.1 | 0.8 ± 0.0 | 0.9 ± 0.1 |
| 24 | 14.6 | 363.1 | 1.0 ± 0.0 | 1.0 ± 0.0 |
| 25 | 16.1 | 365.1 | 0.8 ± 0.0 | 0.9 ± 0.1 |
| 27 | 16.4 | 371.1 | 0.6 ± 0.0 | 0.5 ± 0.1 |
| 28 | 17.1 | 371.1 | 0.7 ± 0.1 | 0.6 ± 0.1 |
| 29 | 21.8 | 376.1 | 1.1 ± 0.0 | 1.0 ± 0.1 |
| 30 | 25.4 | 376.2 | 0.2 ± 0.1 | 0.3 ± 0.0 |
| 31 | 16.5 | 377.1 | 0.9 ± 0.0 | 1.0 ± 0.1 |
| 32 | 21.4 | 379.1 | 0.6 ± 0.1 | 0.6 ± 0.1 |
| 33 | 6.3 | 380.1 | 0.8 ± 0.0 | 0.8 ± 0.0 |
| 34 | 17.5 | 389.1 | 0.7 ± 0.0 | 0.6 ± 0.0 |
| 35 | 24.5 | 389.1 | 0.7 ± 0.0 | 0.7 ± 0.0 |
| 36 | 16.1 | 391.1 | 0.8 ± 0.1 | 0.9 ± 0.1 |
| 37 | 17.0 | 391.1 | 1.1 ± 0.0 | 1.0 ± 0.0 |
| 38 | 18.4 | 391.1 | 0.9 ± 0.2 | 0.9 ± 0.1 |
| 39 | 21.1 | 391.1 | 0.9 ± 0.0 | 0.9 ± 0.0 |
| 40 | 27.9 | 401.2 | 1.8 ± 0.2 | 1.9 ± 0.0 |
| 41 | 28.5 | 403.2 | 1.7 ± 0.0 | 1.8 ± 0.3 |
| 42 | 29.1 | 403.1 | 0.7 ± 0.2 | 0.8 ± 0.1 |
| 43 | 16.9 | 407.1 | 0.7 ± 0.0 | 0.9 ± 0.0 |
| 44 | 17.2 | 407.1 | 0.9 ± 0.0 | 0.8 ± 0.0 |
| 45 | 23.3 | 412.1 | 0.5 ± 0.0 | 0.5 ± 0.0 |
| 46 | 17.2 | 415.1 | 0.5 ± 0.1 | 0.6 ± 0.0 |
| 47 | 16.7 | 416.1 | 0.9 ± 0.1 | 0.9 ± 0.1 |
| 48 | 30.2 | 417.2 | 0.7 ± 0.0 | 0.7 ± 0.0 |
| 49 | 15.8 | 419.1 | 1.1 ± 0.1 | 1.2 ± 0.1 |
| 50 | 18.9 | 419.1 | 0.7 ± 0.0 | 0.9 ± 0.1 |
| 51 | 27.6 | 419.2 | 0.8 ± 0.0 | 0.9 ± 0.1 |
| 52 | 5.8 | 420.1 | 0.8 ± 0.0 | 0.8 ± 0.0 |
| 53 | 11.9 | 423.1 | 0.9 ± 0.2 | 0.9 ± 0.1 |
| 54 | 12.7 | 423.1 | 0.7 ± 0.2 | 0.8 ± 0.0 |
| 55 | 17.1 | 423.1 | 0.9 ± 0.1 | 0.8 ± 0.0 |
| 56 | 26.5 | 423.1 | 1.5 ± 0.0 | 1.5 ± 0.0 |
| 57 | 18.1 | 428.1 | 0.8 ± 0.1 | 0.6 ± 0.1 |
| 58 | 17.9 | 429.1 | 0.9 ± 0.0 | 0.9 ± 0.2 |
| 60 | 33.6 | 429.2 | 0.5 ± 0.0 | 0.6 ± 0.0 |
| 61 | 19.8 | 432.1 | 0.8 ± 0.1 | 0.6 ± 0.1 |
| 62 | 16.5 | 433.1 | 0.5 ± 0.1 | 0.8 ± 0.2 |
| 63 | 17.2 | 433.1 | 0.6 ± 0.0 | 0.6 ± 0.1 |
| 64 | 19.1 | 433.1 | 0.5 ± 0.0 | 0.7 ± 0.0 |
| 65 | 20.7 | 433.1 | 1.2 ± 0.1 | 1.3 ± 0.2 |
| 66 | 22.3 | 433.1 | 0.7 ± 0.1 | 0.8 ± 0.1 |
| 67 | 16.4 | 434.1 | 1.1 ± 0.1 | 1.0 ± 0.0 |
| 68 | 16.5 | 435.1 | 0.7 ± 0.0 | 0.7 ± 0.2 |
| 69 | 17.3 | 435.1 | 0.7 ± 0.1 | 0.7 ± 0.2 |
| 70 | 19.2 | 437.1 | 0.6 ± 0.1 | 0.7 ± 0.1 |
| 71 | 21.4 | 444.1 | 0.6 ± 0.1 | 0.6 ± 0.0 |
| 72 | 33.8 | 447.2 | 0.7 ± 0.1 | 0.6 ± 0.0 |
| 73 | 16.2 | 448.2 | 0.9 ± 0.2 | 0.8 ± 0.2 |
| 74 | 19.2 | 449.2 | 0.7 ± 0.1 | 0.9 ± 0.2 |
| 75 | 14.2 | 450.1 | 0.6 ± 0.1 | 0.7 ± 0.1 |
| 76 | 36.6 | 453.2 | 0.5 ± 0.0 | 0.6 ± 0.1 |
| 77 | 27.9 | 455.1 | 1.0 ± 0.2 | 1.1 ± 0.0 |
| 78 | 5.9 | 459.1 | 0.8 ± 0.2 | 0.7 ± 0.0 |
| 79 | 24.5 | 459.2 | 0.7 ± 0.1 | 0.8 ± 0.0 |
| 80 | 26.0 | 461.2 | 1.6 ± 0.2 | 1.3 ± 0.2 |
| 81 | 16.7 | 462.1 | 0.8 ± 0.0 | 0.9 ± 0.1 |
| 82 | 20.9 | 462.2 | 0.5 ± 0.0 | 0.6 ± 0.1 |
| 83 | 19.8 | 472.1 | 0.6 ± 0.0 | 0.7 ± 0.1 |
| 84 | 7.1 | 477.2 | 0.7 ± 0.1 | 0.9 ± 0.2 |
| 85 | 15.0 | 478.1 | 0.5 ± 0.1 | 0.5 ± 0.0 |
| 86 | 31.1 | 482.2 | 1.1 ± 0.1 | 1.2 ± 0.2 |
| 87 | 16.1 | 490.1 | 0.8 ± 0.2 | 0.7 ± 0.1 |
| 88 | 17.2 | 490.1 | 0.6 ± 0.1 | 0.6 ± 0.1 |
| 89 | 18.2 | 490.1 | 0.8 ± 0.1 | 0.7 ± 0.0 |
| 90 | 11.2 | 491.2 | 0.7 ± 0.1 | 0.9 ± 0.2 |
| 91 | 18.6 | 506.2 | 1.0 ± 0.1 | 0.9 ± 0.1 |
| 92 | 21.2 | 506.2 | 0.8 ± 0.2 | 0.8 ± 0.0 |
| 93 | 27.1 | 528.2 | 0.8 ± 0.0 | 1.2 ± 0.1 |
| 94 | 25.5 | 529.2 | 0.9 ± 0.1 | 0.8 ± 0.1 |
| 95 | 17.3 | 533.2 | 0.7 ± 0.1 | 0.8 ± 0.0 |
| 97 | 14.7 | 546.2 | 0.5 ± 0.0 | 0.6 ± 0.1 |
| 98 | 14.0 | 549.2 | 1.1 ± 0.1 | 1.0 ± 0.1 |
| 99 | 16.9 | 551.2 | 0.6 ± 0.1 | 0.8 ± 0.2 |
| 100 | 18.0 | 563.2 | 1.2 ± 0.1 | 1.4 ± 0.1 |
| 101 | 18.4 | 577.2 | 0.6 ± 0.1 | 0.8 ± 0.1 |
| 102 | 20.1 | 577.2 | 1.1 ± 0.2 | 1.0 ± 0.3 |

Figure S1. The eﬀects of TCEP (A) and BQB (B) contents on the BQB derivatization. *m/z* 261, 377, 379, 401, 429 at retention times of 4.7, 16.5, 21.4, 27.9, 33.6 min, respectively.


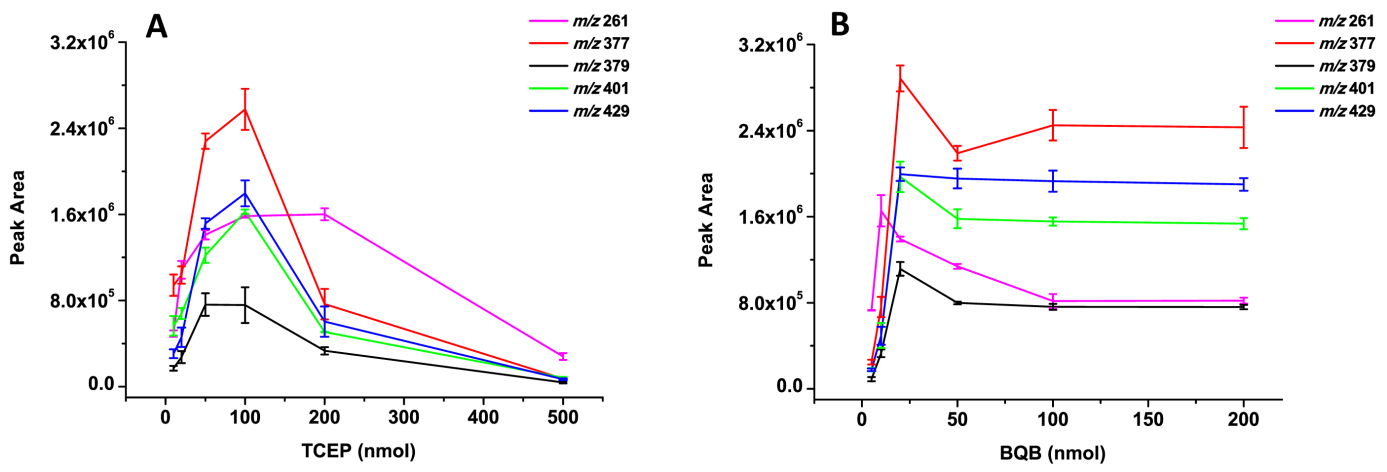


Figure S2. BQB and BQB-*d_7_* labeled cancer urines analyzed by IL-LC-DPI-MS. A, esophagus cancer; B, gastric cancer; C, lymph cancer; D, lung cancer.


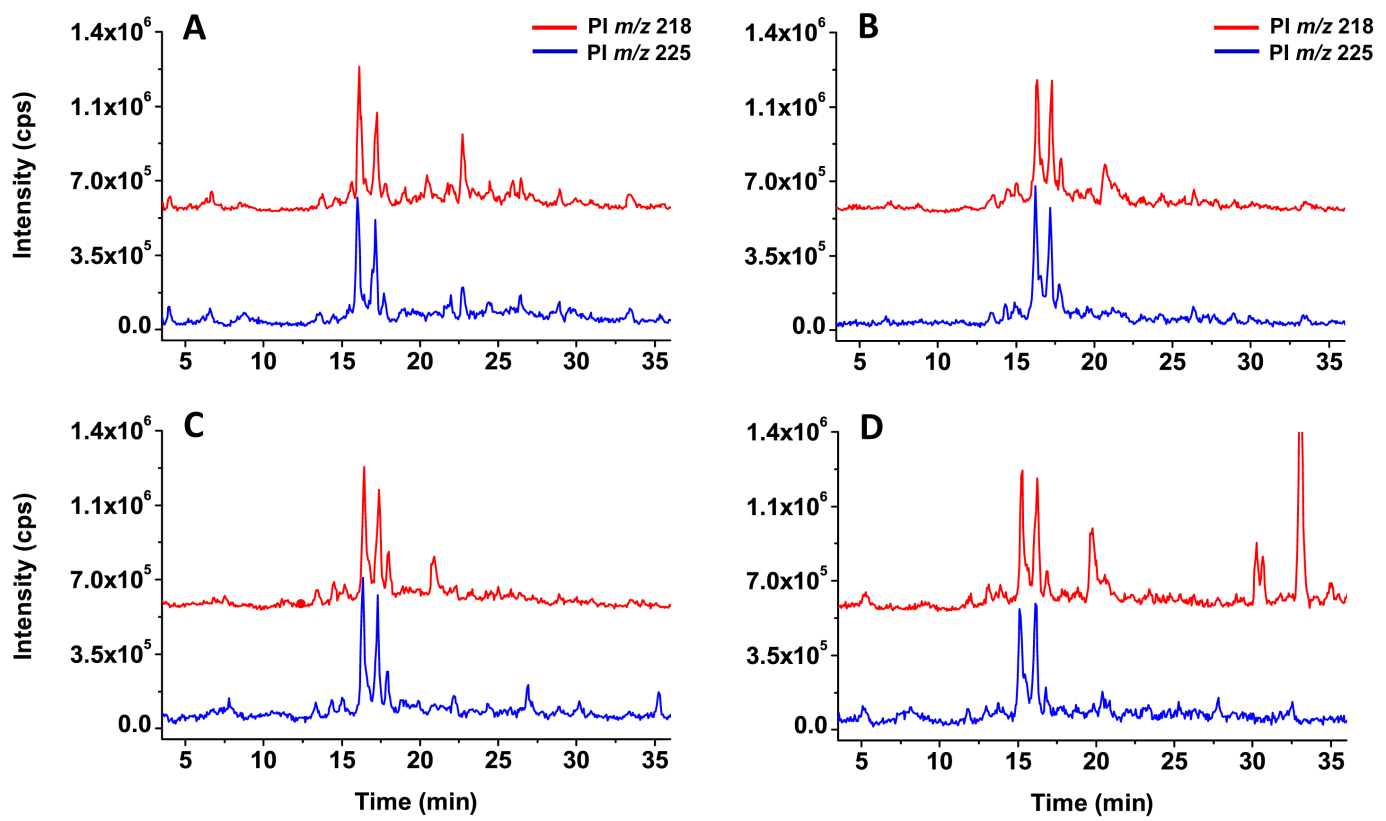


Figure S3. Comparison of the retention time of BQB and BQB-*d_7_* labeled pantetheine standards (0.05 μmol/L, 5 μL) with pantetheine in urine by LC-MRM-MS analysis. (A) BQB labeled pantetheine; (B) BQB-*d_7_* labeled pantetheine.


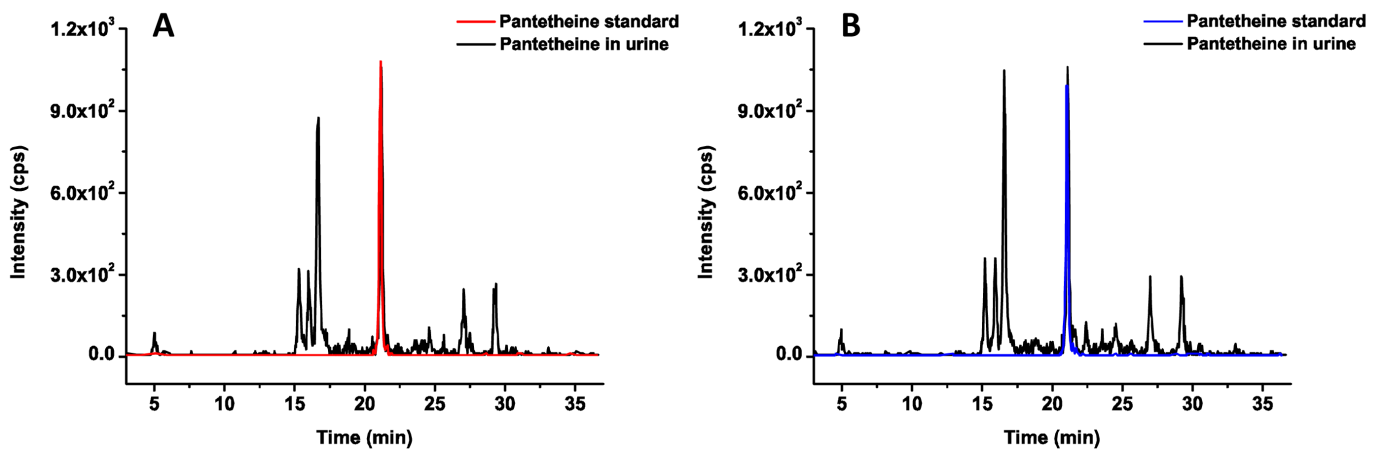


Figure S4. Comparison of the MS/MS spectra of BQB and BQB-*d_7_* labeled pantetheine standards with pantetheine in urine by MS/MS analysis. (A) BQB labeled pantetheine standard; (B) BQB-*d_7_* labeled pantetheine standard; (C) BQB labeled pantetheine in urine; (D) BQB-*d_7_* labeled pantetheine in urine.


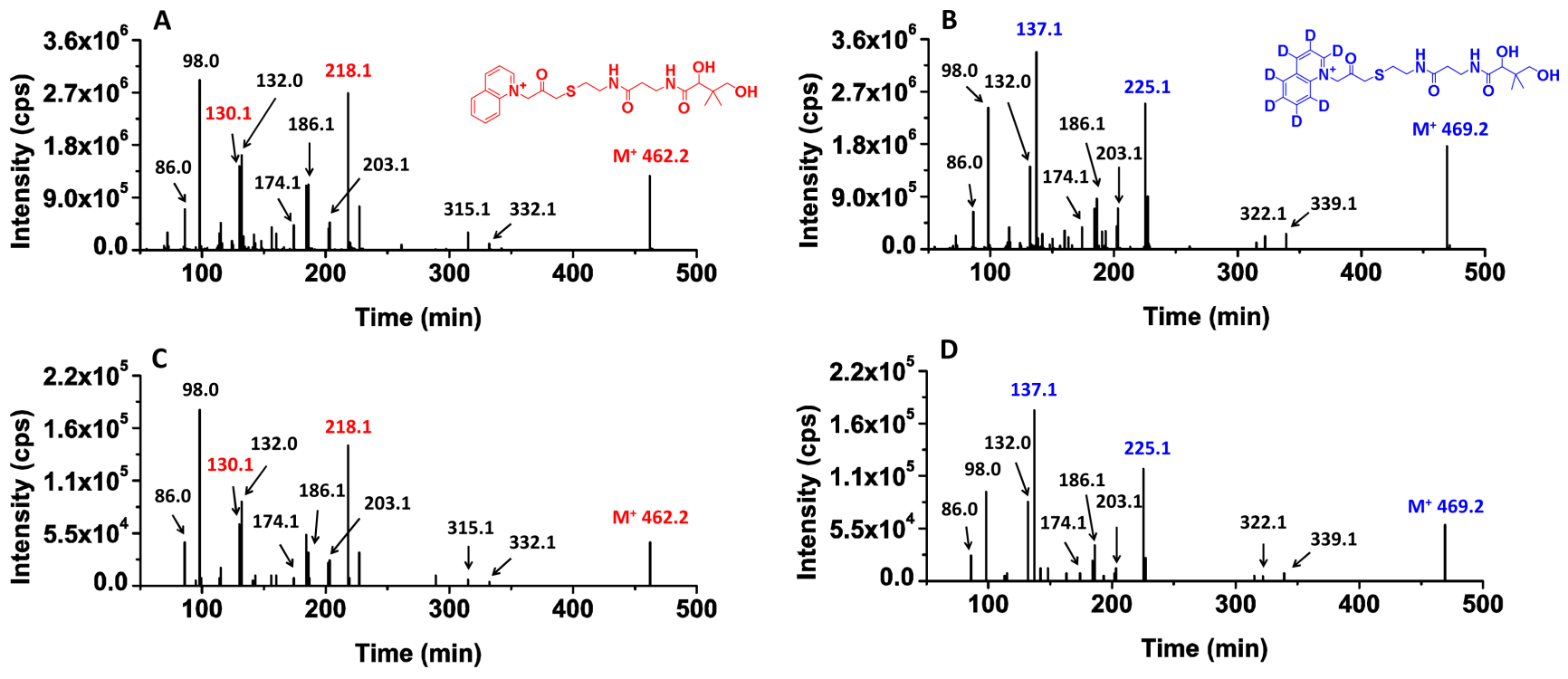


Figure S5. The proposed structures of product ion derived from MS/MS analysis of pantetheine.


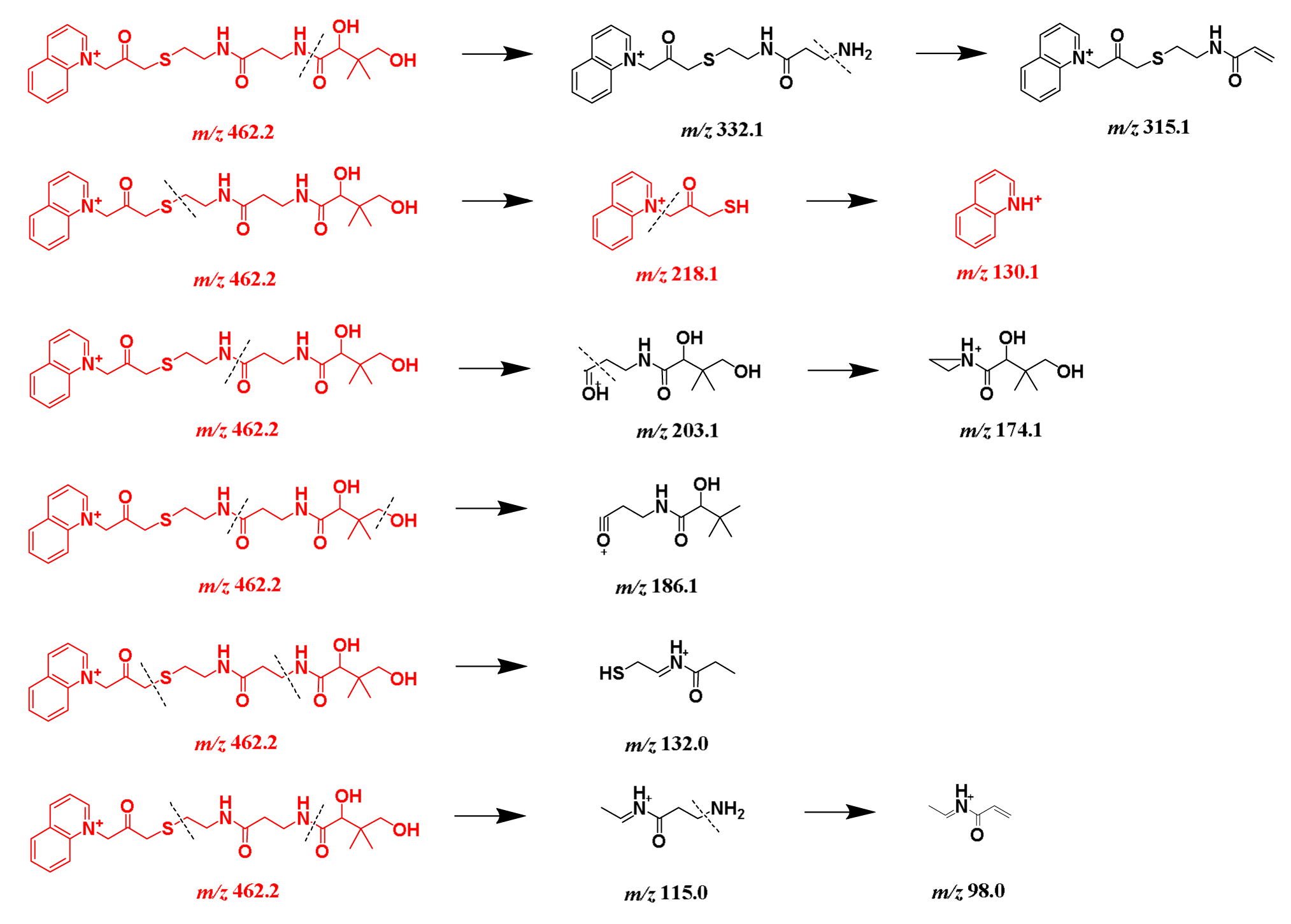


Figure S6. Comparison of LC-DPI-MS with LC-MRM-MS method. A, Extracted ion chromatograms of BQB labeled ion at *m/z* 450 under DPI method; B, Extracted ion chromatograms of BQB labeled ion at *m/z* 461 under DPI method; C, Extracted ion chromatograms of BQB labeled ion at *m/z* 450 under MRM method; D, Extracted ion chromatograms of BQB labeled ion at *m/z* 461 under MRM method.


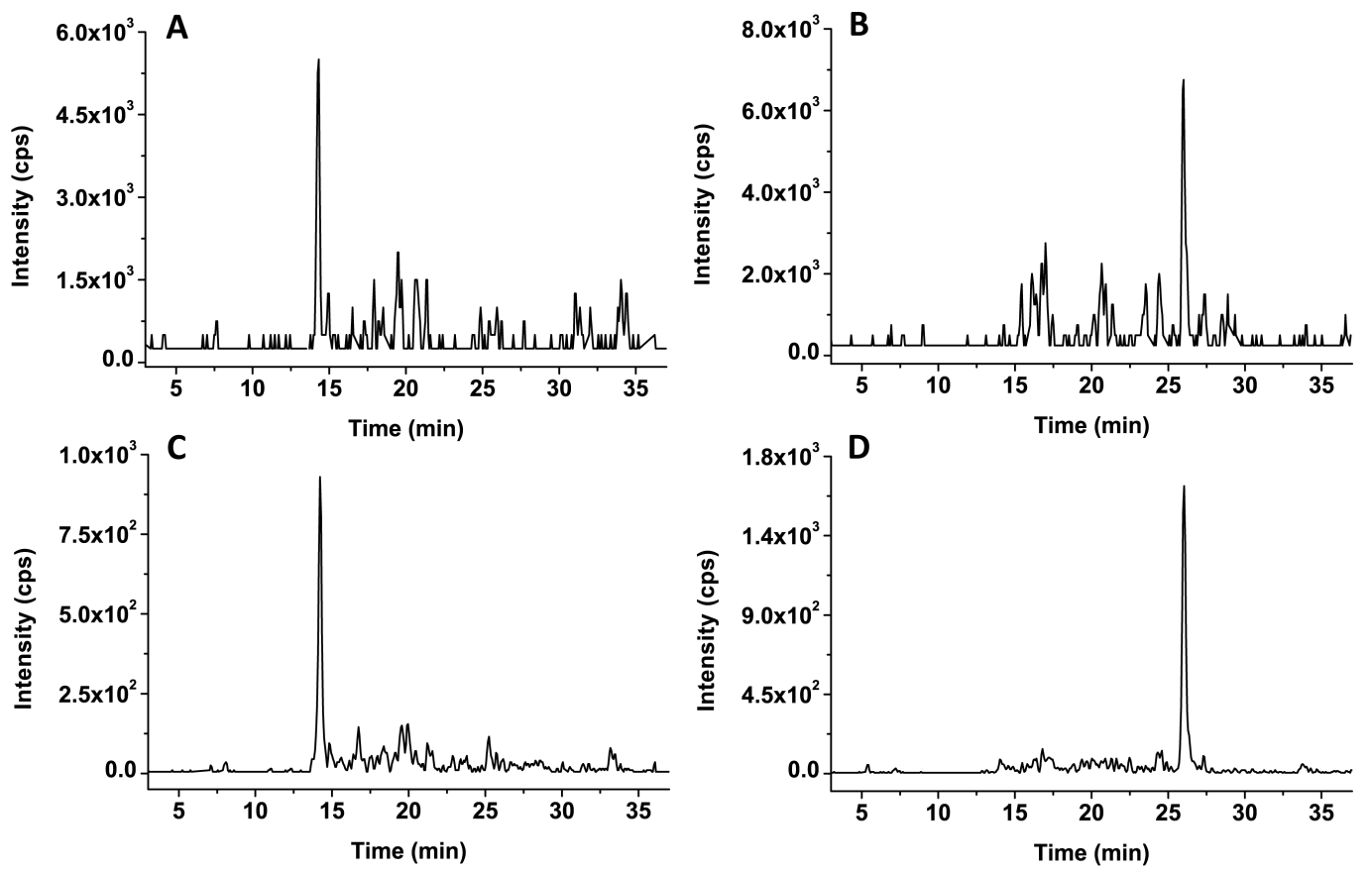


Figure S7. The regression line of the measured peak area ratios versus the concentration ratios (1:10, 1:5, 1:2, 1:1, 2:1, 5:1, 10:1) of BQB/BQB-*d_7_* labeled peak pairs with high intensities from urine.


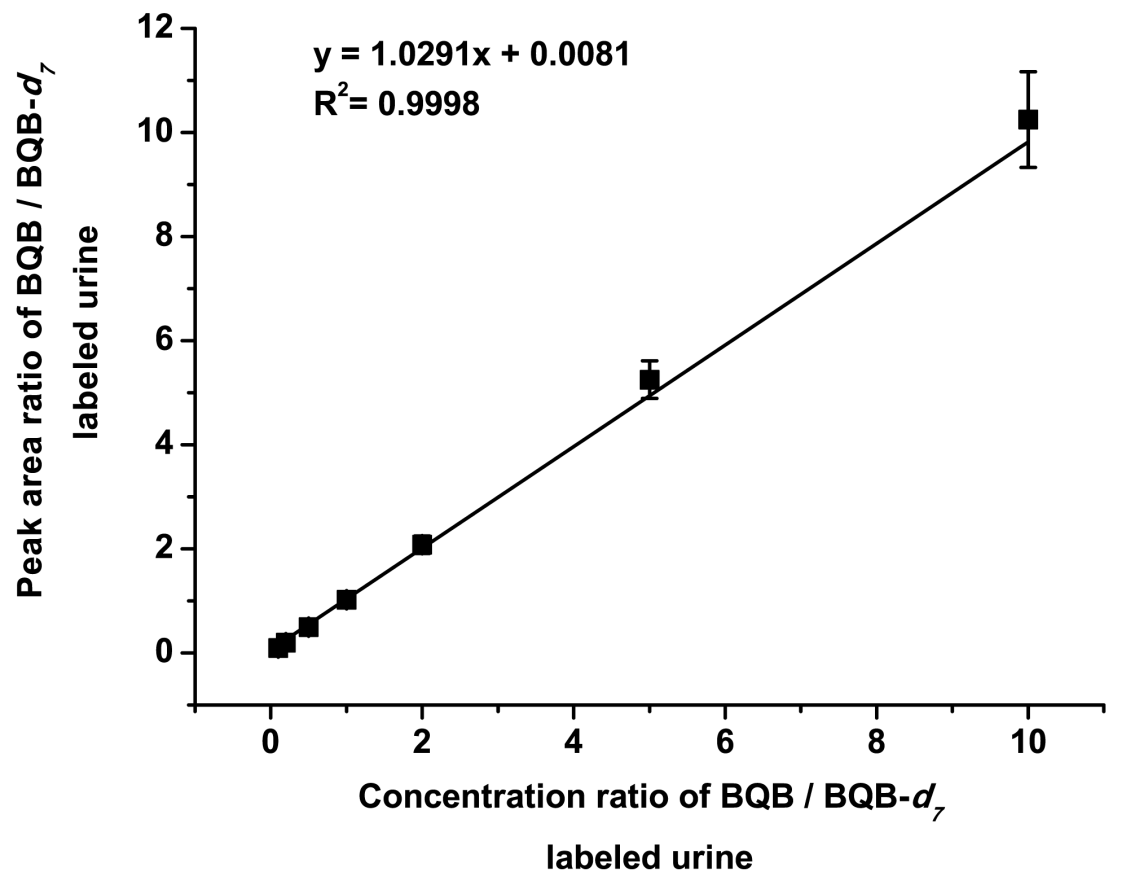

Supplement: Supplementary Information [file srep21433-s1.doc]
